# The Polish COVID Stress Scales: Considerations of psychometric functioning, measurement invariance, and validity

**DOI:** 10.1371/journal.pone.0260459

**Published:** 2021-12-01

**Authors:** Katarzyna Adamczyk, D. Angus Clark, Julia Pradelok

**Affiliations:** 1 Faculty of Psychology and Cognitive Science, Adam Mickiewicz University, Poznań, Poland; 2 Department of Psychiatry, University of Michigan, Ann Arbor, Michigan, United States of America; Universiti Sains Malaysia, MALAYSIA

## Abstract

The COVID Stress Scales (CSS) were developed to measure stress in response to the COVID-19 pandemic. To further investigate the psychometric properties of the CSS, we used data collected in Poland across two waves of assessment (N = 556 at T1 and N = 264 at T2) to evaluate the factor structure, reliability (at the item and scale level), measurement invariance (across the Polish and Dutch translations of the CSS, and time), over time stability, and external associations of the Polish-language version of the CSS (CSS-PL). Overall, results suggest that the CSS-PL is psychometrically robust, largely invariant across the countries and time-lags considered. The CSS-PL was also positively related to other measures of COVID-19 fear, health anxiety, obsessive compulsive symptoms, anxiety, depression, and intent to receive a COVID-19 vaccine. This study thus provides considerable information about the CSS’s items and scales, and lays the foundation for future investigations into COVID stress across time and different populations.

## Introduction

The coronavirus disease 2019 (COVID-19) pandemic has been one of the largest, most widespread pandemics since the end of World War 2 [1, 2]. The COVID-19 pandemic, by nature of its scope and complexity, represents a psychosocial stressor [3] that is distinct from other natural disasters—such as earthquakes or tsunamis—and previous epidemics such as severe acute respiratory syndrome (SARS), Middle East respiratory syndrome (MERS) and Ebola [4]. The current pandemic has disrupted healthcare systems, political dynamics, economic stability, and social functioning around the world [4]. The COVID-19 pandemic has also negatively affected mental health and well-being in a variety of ways [1, 5–7]. One specific psychological consequence of COVID-19 may be the emergence of a multidimensional stress response linked to the pandemic. This has been termed the COVID Stress Syndrome, and is measured by the COVID Stress Scale (CSS) [7, 8].

Given the global nature of the COVID-19 pandemic we anticipate an increasing amount of cross-cultural research into its psychological consequences. This research will require psychometrically vetted instruments that can be used and linked across diverse countries and populations, however. That is, researchers will face the question of whether the COVID Stress Syndrome originally identified in samples from the United States and Canada [8] can also be identified in other populations, and whether the CSS functions similarly across different cultural and national contexts. Therefore, it is critical to both determine that different language versions of the CSS are psychometrically robust within populations, and also that measurement invariance largely holds across different populations.

The CSS was originally developed using English speaking, North American samples [8] but has already been translated into a number of languages, including Albanian, Chinese, French, Lithuanian, Malay, Marathi, Hindi, Persian, Portuguese (adapted for adolescents), Serbian, Spanish, Swedish, Turkish, and Urdu (https://coronaphobia.org/professional-resources/). However, no study has explored the psychometric properties of a Polish language version of the CSS in a Polish sample. Therefore, the overarching goal of the current study was to develop and evaluate the Polish language version of the CSS (CSS-PL) in order to further facilitate the global study of psychological responses to the COVID-19 pandemic. The first case of SARS-CoV-2 in Poland was confirmed on March 3, 2020, and since then 2, 904, 631 people have been infected, and 75, 601 have died from infections (as of September 2021; www.gov.pl). In the face of the pandemic, the Polish government has introduced a number of restrictions in order to try and stop the spread of the virus [9, 10]. Given the impact that COVID-19 has had on the people of Poland, it is important that there are well-vetted instruments available for measuring the psychological impact of the pandemic on this population.

## The COVID Stress Scales (CSS)

The CSS was developed in order to provide a multifaceted assessment of COVID‐19‐related distress, though there was also the intention to craft the CSS such that it could be adapted for use during future pandemics [8]. The calibration data for the original CSS was collected between March 21 and April 1, 2020 from individuals in the United States and Canada. Based on this data Taylor and colleagues [8] determined that a five-factor structure was the most reasonable characterization of the CSS. Specifically, the 36-item CSS includes the following five subscales: (1) Danger and Contamination Fears (DAN), (2) Socioeconomic Consequences Fears (SEC), (3) Xenophobic Fears (XEN), (4) Traumatic Stress Symptoms (TSS), and (5) Compulsive Checking and Reassurance seeking (CHE). Each scale includes six items, with the exception of the Danger and Contamination Fears scale which contains 12 (though danger and contaminations constructs may be measured separately if necessary) [8]. These five scales tend to be strongly intercorrelated, which has been interpreted as providing evidence for the existence of a coherent syndrome termed the “COVID Stress Syndrome” [8], the core of which is the fear of becoming infected with COVID-19 [7]. The five individual scales can be aggregated for assessing the presence of the COVID Stress Syndrome.

The CSS scales generally demonstrate good-to-excellent internal-consistency reliability [8]. There is also evidence for temporal stability in the CSS over a 4-week interval [11]. The CSS is also positively associated with anxiety, depression, health anxiety, and checking and washing as symptoms characteristic of obsessive compulsive disorder (OCD) [8]. Notably, correlations with anxiety tend to be higher than those with depression. Furthermore there is evidence that the COVID Xenophobia scale is more strongly related to negative attitudes towards immigrants than the other scales [8].

## The present study

The first aim of the present study was to develop a Polish version of the CSS. This involved translating the original CSS into the Polish-language, comparing the Polish translation of the CSS to the original English CSS in a small sample of bilingual university students, and examining the factor structure of the initial CSS-PL in a small online pilot sample of Polish participants.

Once the CSS-PL was developed, the second aim of the study was to characterize the CSS-PL’s psychometric properties in terms of factor structure. As a part of this, and to extend past work with the CSS, in the current study we evaluated measurement invariance (MI) in the CSS across two countries. Specifically, we tested MI across the Polish and Dutch CSS translations [2], and then characterizing the item and test properties of the Polish and Dutch CSS translations using item response models. Measurement invariance (MI) refers to the issue of if the item parameters in a measurement model differ meaningfully across groups, and/or time. Notably, MI is referred to under the label Differential Item Functioning (DIF) in the Item Response Theory (IRT) literature. Although we apply item response models in the analyses here we use the term MI throughout the manuscript as it tends to be more frequently encountered in the psychological literature [12]. When testing MI the focus is specifically on the equivalence of item discrimination parameters (*a*; discrimination parameter are analogous to factor loadings), as well as item location parameters (*b*). Location parameters are similar to indicator intercepts and reflect the level of the latent trait needed to endorse a higher category on a specific item. There are multiple ways to express and parameterize item location parameters, and here we specifically refer to item difficulty parameters, which capture the point at which the probability of endorsing a higher category reaches 50%.

MI is crucial to consider because the presence of non-invariance (NI) effectively puts groups on different metrics, even though the latent factors are ostensibly measuring the same construct with the same instrument. This renders group comparisons (or comparisons over time)—either of means or associations with external variables—potentially invalid as any observed differences could merely be due to NI (i.e., a methodological artifact). Importantly, the presence or absence of MI has no inherent implications for whether there are true group/time differences on the latent construct, but if NI is identified, observed differences may not be accurate until NI has been addressed. Addressing NI could involve revising items, removing them from the assessment, ignoring it if largely inconsequential, or adjusting scores by incorporating NI into the measurement model [13].

The third aim of the present study was to consider MI and the stability of scores in the CSS-PL over a one-month interval. In the current investigation, we used a 4-week interval to be consistent with recent work on the CSS [11]. Furthermore, although a 1 month interval would generally be considered short for assessing measurement invariance over time, given the dynamic nature of the COVID-19 pandemic in terms of pathogenesis, diagnosis, prognosis, and treatment options [14], it is worth starting by considering changes over relatively brief lags [14]. Indeed, testing MI is an essential element of analyzing change over time [15], and will be critical in future investigations in determining whether temporal changes in the COVID Stress Syndrome are the result of true increases or decreases in the construct, or just artifacts of measurement.

The fourth aim of the present study was examine convergent, divergent, and criterion associations between the CSS-PL and a number of other variables. In order to highlight the similarity of the CSS-PL with the original CSS we focused here on associations between the CSS-PL and many of the variables that have previously been considered in relation to the CSS [8]. We specifically considered associations between the CSS-PL and (a) health anxiety as measured by the Short Health Anxiety Inventory (SHAI) [16]; (b) obsessive compulsive and obsessive compulsive washing as measured by the Obsessive Compulsive Inventory-Revised (OCI-R) [17]; (c) anxiety and depression as measured by the Patient Health Questionnaire-4 (PHQ-4) [18]; (d) xenophobia as measured by the Political Beliefs Questionnaire (PBQ) [19]; and (e) social desirability as measured by the Social Desirability Scale (SDS-17) [20]. We also considered how strongly the CSS-PL was related to a similar assessment of COVID related stress—the Fear of COVID-19 Scale (FCV-19S) [21]—and, to examine divergent associations, the extent to which the CSS-PL was associated with impulsive behavior measured by the Impulsive Behavior Scale Short Version (SUPPS-P) [22]. Finally, we examined concurrent and lagged (across a 1-month interval) associations between the CSS-PL and intent to receive a COVID-19 vaccine.

We emphasized intent to be vaccinated as an important outcome since hesitancy toward COVID-19 vaccination may constitute a major obstacle in achieving herd immunity [23], and the issue of vaccination has been intensively investigated in the recent literature [23–25]. Although links between the CSS and intent to receive a COVID-19 vaccine was not examined in the original study [8], prior research on the H1N1 influenza (also known as swine flu) pandemic [26], as well as other research on the COVID-19 pandemic [27], is instructive. In these instances the protective motivation theory (PMT) has been invoked. This theory postulates that protection motivation, i.e., “a behavioral intention to perform a maladaptive or adaptive behavior” [26: 816] is a result of two factors—threat and coping appraisals [BISH]. Indeed, there is evidence that higher general anxiety is associated with increased likelihood of protective behaviors [26], and that threat-appraisal of potential infection is also associated with the motivation for social distancing in the COVID-19 pandemic [27]. Based on this past work and the protective motivation theory we expected that stress and anxiety as measured by the Polish version of the CSS at Time 1 would be positively related to intent to have a COVID-19 vaccine at T1, and after four weeks later at T2.

## Methods

### Participants and procedure

All questionnaires were administered to the Polish sample via an online survey between March 29, 2021 and April 29, 2021. During this period there was a significant increase in COVID-19 infections in Poland, and several new safety measures were imposed, including the closure of big-box stores and shopping malls, limits on people attending religious worship, closure of hair and beauty salons, nurseries and kindergartens (www.gov.pl).

The data collection protocol was approved by the Research Ethics Committee at Faculty of Psychology and Cognitive Science, Adam Mickiewicz University in Poznań (decision number: 1/02/2021) and all respondents consented prior to beginning the survey. The inclusion criteria required potential participants to be at least 18 years old and provide informed consent. No exclusion criteria were applied in the current study. Compensation for participation was provided in the form of a lottery in which participants could win vouchers ranging from 10 PLN to 25 PLN for an online store. We report how we determined our sample size, all data exclusions, all manipulations, and all measures in the study.

The minimal sample size of the validation study was determined based on an a priori power calculation (https://sample-size.net/correlation-sample-size/). Specifically, to detect small-sized correlation coefficients (.20) with sufficient statistical power (.80) the study would require at least 194 respondents. Further, since our analyses employed the IRT-based methodology that requires a large sample size (N > 500) [28], we allowed a larger reference sample size to be recruited to ensure the requirements of IRT analysis.

The Polish sample originally consisted of 556 respondents (62.10% women; aged 18–85 years with an average age of 43.55). Among 556 participants at T1, 19.10% (*n* = 106) had had COVID-19, whereas 274 participants (49.30%) participants definitively endorsed not having had coronavirus. At the second wave of assessment 264 participants from the first assessment completed the CSS-PL (attrition rate of 52.50%). In turn, the Dutch sample come from Vos and colleagues’ study [2] and includes 382 Dutch individuals (45.80% women; aged 19–76 years with an average age of 30.49). The participants in the study by Vos and colleagues [2] were recruited through the Prolific online working platform and the social networks of involved students. The materials were in the Dutch language, and respondents mainly originated from the Netherlands and Belgium. The final sample in the study by Vos and colleagues [2] included 546 participants (*M*_*age*_ = 29.81, *SD* = 10.36). Among the eligible 546 respondents, 382 individuals were from the Netherlands, and 112 were from Belgium. In the present investigation, for IRT analysis that requires larger samples [2], we utilized only the subset of the data collected from the respondents from the Netherlands. For a detailed description of the Dutch sample and the procedures used to collect this sample, see Vos and colleagues [2].

The data for the Polish sample is freely available through the Open Science Framework (https://osf.io/k5234/?view_only=b3a0973e00664747b148f661d67c11d4). The data collected in the Netherlands by Vos and colleagues [2] are also available through the Open Science Framework (https://osf.io/xb865/).

## Measures

***The COVID-Stress Scales*** [8] is a 36-item self-report instrument consisting of five subscales: (1) COVID danger and contamination (12 items); (2) COVID fears about economic consequences (6 items), (3) COVID xenophobia (6 items), (4) COVID traumatic stress symptoms (6 items) and (5) COVID compulsive checking and reassurance seeking (6 items). All the 36 items are rated on a five-point Likert scale from 0 to 4. The CSS was originally developed in English. With the permission of the original authors of the CSS [8], the CSS was translated into the Polish language following the recommendations of the “ISPOR Task Force for Translation and Cultural Adaptation” [29]. A series of pilot studies suggested an effective translation process, and that the CSS-PL functioned similar to the CSS in terms of the metrics used in the original development paper (e.g., factor structure, reliability). Greater detail about the initial translation and evaluation of the CSS-PL can be found in (along with the translation itself; see S1, S2 Files and S1–S3 Tables). The mean scores of each subscale were calculated and used in the analyses.

***The Fear of COVID-19 Scale*** (FCV-19S) [21] includes seven items assessing fear related to the COVID-19. Participants are asked to rate each item using a five-point Likert scale from 1 (*strongly disagree*) to 5 (*strongly agree*). In the present study a Polish translation of the FCV-19S was used, and the Omega coefficient for the scale was .89. The mean score of the FCV-19S was calculated and used in the analyses.

***The Patient Health Questionnaire-4*** (PHQ-4) [18] (Polish translation for derived from the website: https://www.phqscreeners.com/select-screenert). The PHQ-4 includes four items assessing anxiety (2 items) and depression (2 items) over the past two weeks. Participants rate four items a on a four-point Likert scale ranging from 0 (*not at all*) to 3 (*almost every day*). In the current study, the Omega coefficient of the PHQ-4 was .90. The mean score of the PHQ-4 was calculated and used in the analyses.

***The Short Health Anxiety Inventory*** (SHAI) [16] in Polish translation [30] assess health anxiety independently of physical health status. Participants rate items on a four-point scale from 0 to 3 describing their anxiety over the past six months. In this study, the Omega coefficient for the SHAI was .93. The mean score of the SHAI was calculated and used in the analyses.

***The Social Desirability Scale*** (SDS-17) [20] in Polish adaptation [31] includes 16 items assessing social desirability. Respondents rate each statement as either true (1) or false (0) for them. In the current study, the Omega coefficient for the SDS-17 was .75. The mean score of the SDS-17 was calculated and used in the analyses.

***The Obsessive Compulsive Inventory-Revised*** (OCI-R) [17] in Polish adaptation [32] is an 18-item instrument measuring obsessive-compulsive symptoms. Participants rate 18 items using a five-point Likert scale ranging from 0 (*not at all*) to 4 (*extremely*). In the current study, we used items pertaining to compulsive washing (3 items) and checking (3 items). In the current study, the Omega coefficients of the washing and checking scales were .82 and .80, respectively. The mean scores of the OCI-R was calculated and used in the analyses.

***The Political Beliefs Questionnaire*** (PBQ) [19] was designed and validated in Poland to measure religious fundamentalism, xenophobia, and economic beliefs [19]. The PBQ consists of 19 items rated a five-point Likert scale from 1 (*strongly disagree*) to 5 (*strongly agree*). In the current study, xenophobia subscale was used; the Omega coefficient was .91. The mean score of the xenophobia subscale was calculated and used in the analyses.

***The Impulsive Behavior Scale Short Version*** (SUPPS-P) [22] in Polish adaptation [33] assesses different facets of impulsivity, including urgency, premeditation, perseverance, sensation seeking. Items which are rated on a four-point Likert scale from 1 (*strongly agree*) to 4 (*strongly disagree*). In the current study, the sensation seeking subscale (4 items) was used; the Omega coefficient was .87. The mean score of the impulsive behavior subscale was calculated and used in the analyses.

***Demographic information***. Respondents were asked to indicate their age, the gender they identify with the most, highest educational level, place of residence, whether they have been infected by the virus, whether they knew anyone that is/was infected by the virus, if they have received a COVID-19 vaccine, and if they intend to receive a COVID-19 vaccine (rated on a 5-point Likert scale).

## Data analytic strategy

The analytic strategy consisted of five major steps. In the first step we used exploratory structural equation modeling (ESEM) [34] to examine the dimensional structure of the final Polish CSS (CSS-PL). ESEMs were fit in both the Polish (Time 1) and Dutch samples. Based on the CSS’ construction (i.e., 6 distinct scales tapping into an overarching syndrome), results from the original dimensionality assessment, and pilot data on the CSS-PL, single factor, 4-factor, 5-factor, and 6-factor solutions were all specified. In the 5-factor solution the Danger and Contamination scales were specified to target the same factor. In the 4-factor solution the Traumatic Stress and Checking scales were also combined into a single factor. The ESEMs were fit in M*plus* version 8.5 [35] with oblique target rotations using mean and variance adjusted weighted least squares (WLSMV) estimation.

In the second step, we examined the extent to which the CSS demonstrated measurement invariance across the Polish and Dutch translations. Tests of measurement invariance were based on the graded response model [36], and two variants of the improved Wald Test for DIF [37, 38]. The graded response model (GRM) is an IRT model for polytomous items (i.e., items with more than two response categories); the GRM is effectively a categorical confirmatory factor model in the responses to a given CSS item are model as a probabilistic function of the latent trait of interest [39]. For the DIF analysis, all items and item parameters were first simultaneously tested for measurement non-invariance (NI) in an initial sweep. An advantage of this approach is that all items are simultaneously tested for NI; however, it is prone to an inflated false-positive rate [38]. Thus, this was used primarily to identify anchor items, and flag items that might contain NI across groups. More focused, robust tests of NI were subsequently conducted based on these initial results. All items that showed no evidence of NI in the initial sweep were constrained to equality across groups in the subsequent NI model, while all items that exhibited evidence of NI were freely estimated across groups. Additional NI models were then fit in which the item parameters that did not exhibit NI in a prior model were constrained to equality across groups, while those item parameters that did evidence NI were freely estimated. This was done in order to identify the most parsimonious multi-group model that accounted for NI, and because more anchor items increases the power and robustness of NI tests. The item response models and NI tests were conducted in flexMIRT version 3.6 [40] using full information maximum likelihood estimation with the supplemented expectation maximization (SEM) algorithm [37].

In the third step, we considered the reliability of the CSS-PL at the item the scale level. Item discrimination values, and scale information functions, from the final graded response model identified in the previous step (i.e., the most justifiably constrained model across groups) were used to help characterize the reliability of the CSS-PL. Coefficient omegas (*ω*) [41] were also computed for each scale of the CSS-PL in order to provide a single value, holistic summary of scale reliability.

In the fourth step, we examined measurement invariance in the CSS-PL from Time 1 to Time 2, as well as rank-order stability over time. Tests of measurement invariance here followed the same procedure outlined in step two, however, instead of comparing the Polish and Dutch samples, the comparisons were between the Polish sample at times 1 (reference assessment) and 2 (focal assessment).

In the last step, we assessed the convergent, discriminant, and criterion validity of the CSS-PL by calculating Pearson’s *r* correlations using the SPSS v. 27.00. Convergent associations were examined between the CSS-PL and measures of anxiety-related traits (the SHAI), obsessive-compulsive symptoms (the OCI-R), as well as COVID-19-related fear (the FCV-19S). These correlates have notably been included in other examinations of the CSS and its translations [8, 11]. Discriminant associations were examined between current anxiety (the PHQ-4), depression (the PHQ-4), xenophobia (the PBQ), social desirability (the SDS-17) and sensation seeking (the Short UPPS-P). Concurrent and prospective criterion associations were examined (among unvaccinated participants) between the CSS-PL and intent to receive a COVID-vaccine within Time 1, and from Time 1 to Time 2.

## Results

### Attrition rate

In the first step of analysis, we determined the attrition rate between the first and second assessment after four weeks. The attrition rate was estimated to be 52.50% (292 participants dropped out of the study by T2). On average, participants who did not complete the second assessment were older, less willing to receive a COVID-19 vaccine, and more likely to be unsure about if they knew anyone infected with COVID-19 compared to those who completed the second assessment (see Table 1).

**Table 1:** Sample characteristics of the total sample at T1 and T22 and as a function of attrition rate between T1 and T2.

		W1-W2 Attrition comparisons		
Variable	Total sample at T1	Participants who were at T1 and T2	Participants who were at T1 and dropped at T2	Difference
	(N = 556)	(*n* = 264)	(*n* = 292)	
Age, years				*p* < .001
*M (SD)*	43.55 (14.93)	40.46 (14.10)	46.34 (14.33)	
Range	18–85	19–85	18–80	
Gender, *n* (%)				*p* = .428
Male	203 (36.50%)	89 (33.70%)	114 (39.0%)	
Female	345 (62.10%)	171 (64.80%)	174 (59.60%)	
Other	8 (1.40%)	4 (1.50%)	4 (1.40%)	
Place of residence, *n* (%)				*p* = .059
Village^a^	77 (13.80%)	29 (11.00%)	48 (16.40%)	
City < 25, 000	55 (9.90%)	25 (9.50%)	30 (10.30%)	
City 25,000–50,000	55 (9.90%)	21 (8.00%)	34 (11.60%)	
City 50,000–200,000	101 (18.20%)	44 (16.70%)	57 (19.50%)	
City 200,000–500,000	61 (11.00%)	31 (11.70%)	30 (10.30%)	
City > 500,000	207 (37.20%)	114 (43.20%)	93 (31.80%)	
Highest education, *n* (%)				*p* = .063
Primary education	2 (0.40%)	1 (0.40%)	1 (0.30%)	
Lower secondary education	1 (0.20%)	-	1 (0.3%)	
Vocational education	7 (1.30%)	1 (0.40%)	6 (2.10%)	
Secondary education	97 (17.40%)	38 (14.40%)	59 (20.20%)	
Higher education	398 (71.60%)	193 (73.10%)	205 (70.20%)	
Student	51 (9.20%)	31 (11.70%)	20 (6.80%)	
Employment, *n* (%)				*p* = .221
Unemployed	63 (11.30%)	29 (11.00%)	34 (11.60%)	
Part-time job	18 (3.20%)	10 (3.80%)	8 (2.70%)	
Permanent job	34 (6.10%)	151 (57.20%)	165 (56.60%)	
Own business activity	8 (1.40%)	29 (11.00%)	26 (8.90%)	
Retired	274 (49.30%)	26 (9.80%)	43 (14.70%)	
Disablement pension	67 (12.10%)	7 (2.70%)	5 (1.70%)	
Other forms of employment	92 (16.50%)	12 (4.50%)	11 (3.80%)	
Infected by the coronavirus?, *n* (%)				*p* = .178
Yes	106 (19.10%)	56 (21.20%)	50 (17.10%)	
No	274 (49.30%)	135 (51.10%)	139 (47.60%)	
I do not know	120 (21.60%)	47 (17.80%)	73 (25.00%)	
I had a suspicion of the coronavirus	56 (10.10%)	26 (9.80%)	30 (10.30%)	
Know someone infected?				*p* = .014
Yes	427 (76.80%)	211 (79.90%)	216 (74.00%)	
No	92 (16.50%)	44 (16.70%)	48 (16.40%)	
Unsure	37 (6.70%)	9 (3.40%)	28 (9.60%)	
Having a COVID-19 vaccine?				*p* = .220
Yes	194 (34.90%)	99 (37.50%)	95 (32.50%)	
No	362 (65.10%)	165 (62.50%)	197 (67.50%)	
Intent to have a COVID-19 vaccine	3.62 (1.65)	4.19 (1.36)	3.14 (1.72)	*p* < .001
Total CSS-PL score	1.14 (0.67)	1.16 (0.65)	1.12 (0.68)	*p* = .405
FCV-19S score	2.36 (0.98)	2.42 (0.96)	2.29 (0.99)	*p =* .115

*Note*. In the Polish law, a village is a settlement unit with compact or scattered buildings and existing agricultural or related service or tourist functions, which does not have urban rights or city status (https://stat.gov.pl/metainformacje/slownik-pojec/pojecia-stosowane-w-statystyce-publicznej/1308,pojecie.html).

### Factor structure of the CSS in the Polish and Dutch samples

Model fit for the 1, 4, 5, and 6 factor ESEM solutions in the Polish and Dutch samples are presented in Table 2. The factor loadings associated with the various solutions are presented in S4–S7 Tables.

**Table 2:** Model fit for exploratory structural equation models across Polish and Dutch samples.

	χ^2^	df	*p*	RMSEA	SRMR	CFI	TLI
**Polish sample**							
1-Factor	8769.05	594	< .01	.157	.158	.768	.754
4-Factor	2541.78	492	< .01	.087	.045	.942	.926
5-Factor	1540.28	460	< .01	.065	.029	.969	.958
6-Factor	1091.64	429	< .01	.053	.022	.981	.972
**Dutch sample**							
1-Factor	4480.75	594	< .01	.131	.135	.786	.773
4-Factor	1521.21	492	< .01	.074	.055	.943	.927
5-Factor	1021.00	460	< .01	.057	.040	.969	.958
6-Factor	767.49	429	< .01	.045	.031	.981	.973

χ^2^ = chi-square value for chi square exact test of model fit; df = model degrees of freedom for chi-square exact test of model fit; *p* = p-value for chi-square exact test of model fit. Models fit using weighted least squares with mean and variance adjustment (WLSMV) estimation and targeted oblique rotation. The rotation targets for items not associated with a factor were set to 0.

**Fig 1:**
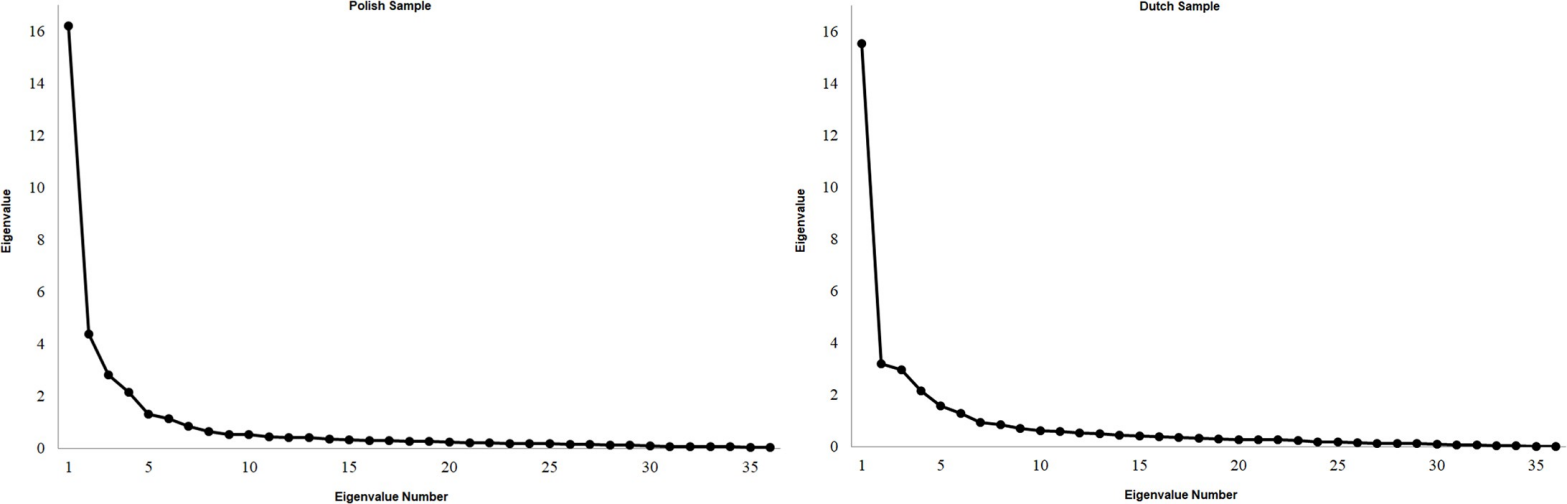
Scree plots for COVID Stress Scales item factor analyses in the Polish and Dutch samples.

Scree plots for COVID Stress Scales item factor analyses in the Polish and Dutch samples are provided in Fig 1.

The 4, 5, and 6 factor solutions all fit well on the basis of conventional fit thresholds (i.e., RMSEA/SRMR < .08; CFI/TLI > .90) [42], suggesting that these three solutions represent reasonable characterizations of the CSS in both samples. In both samples the pattern of factor loadings in the 6-factor solution was consistent with the original six scales of the CSS. Consistent with the 5-factor solutions reported by Taylor and colleagues [8], in the 5-factor solutions the Danger and Contamination scales loaded onto a single factor. However, in both the Polish and Dutch samples the Traumatic Stress and Checking scales also appeared to cohere into a single factor in the 5-factor solutions.

Four factors thus appeared adequate to characterize the CSS in both the Polish and Dutch samples, however the 5-factor solution was ultimately retained for the remainder of the analyses. This was done to remain consistent with the original results provided by Taylor and colleagues [8], as well as with other non-English language versions of the CSS [11, 43, 44]; that is, most applications of the CSS use the 5-factor solution, and so it was retained here to maintain conceptual coherence across the relevant literature. Also, the 5-factor solution still appeared reasonable in the Polish and Dutch samples, even if it was not the most parsimonious (it simply involved splitting the Traumatic Stress and Checking Scales into distinct factors). As a follow-up to the ESEMs a correlated factors model was fit based on the 5-factor solution. The results for this model can be found in S8 Table. Items loaded strongly on their associated factors, with average factor loadings of λ = .83 for the Polish sample, and λ = .82 for the Dutch sample.

### Measurement invariance in the CSS across Poland and the Netherlands

The results from the MI tests are presented in Table 3.

**Table 3:** Results from COVID Stress Scales measurement invariance tests across Polish and Dutch samples.

	All Items			Candidate Items 1			Candidate Items 2			Candidate Items 3		
	I χ^2^	*a* χ^2^	*b* χ^2^	I χ^2^	*a* χ^2^	*b* χ^2^	I χ^2^	*a* χ^2^	*b* χ^2^	I χ^2^	*a* χ^2^	*b* χ^2^
DC-1	3.50	.50	3.10	--	--	--	--	--	--	--	--	--
DC-2	5.30	2.10	3.20	--	--	--	--	--	--	--	--	--
DC-3	**87.90**	**22.80**	**65.10**	**73.70**	**22.90**	**50.80**	**191.20**	**24.90**	**166.20**	**163.70**	**7.20**	**156.60**
DC-4	**14.0**	1.20	**12.90**	**14.40**	1.20	**13.10**	**17.80**	1.50	**16.30**	14.60	--	**14.60**
DC-5	**85.20**	**32.10**	**53.00**	**71.70**	**33.10**	**38.50**	**136.10**	**35.30**	**100.70**	**98.90**	**6.50**	**92.40**
DC-6	5.60	1.90	3.70	--	--	--	--	--	--	--	--	--
DC-7	**13.10**	**7.40**	5.70	**11.10**	**5.80**	5.30	10.60	**4.60**	6.0	--	--	--
DC-8	**13.90**	**7.10**	6.70	7.70	**4.20**	3.50	--	--	--	--	--	--
DC-9	**17.60**	**12.00**	5.60	10.80	**9.40**	1.40	--	--	--	--	--	--
DC-10	**16.10**	**7.60**	8.50	11.00	**4.10**	6.90	--	--	--	--	--	--
DC-11	**14.50**	**3.10**	**11.40**	10.50	1.30	9.10	--	--	--	--	--	--
DC-12	4.30	**3.90**	.40	--	--	--	--	--	--	--	--	--
SES-1	3.00	0.00	3.00	--	--	--	--	--	--	--	--	--
SES-2	9.80	2.20	7.70	--	--	--	--	--	--	--	--	--
SES-3	3.40	1.20	2.20	--	--	--	--	--	--	--	--	--
SES-4	2.00	.90	1.10	--	--	--	--	--	--	--	--	--
SES-5	7.20	.50	6.60	--	--	--	--	--	--	--	--	--
SES-6	5.40	.40	5.00	--	--	--	--	--	--	--	--	--
XN-1	**11.50**	.50	**11.00**	**32.50**	1.60	**30.90**	**39.00**	--	**39.00**	--	--	--
XN-2	4.00	.10	3.00	--	--	--	--	--	--	--	--	--
XN-3	2.80	.50	2.30	--	--	--	--	--	--	--	--	--
XN-4	5.40	.20	5.20	--	--	--	--	--	--	--	--	--
XN-5	**15.80**	2.60	**13.20**	**32.60**	**6.00**	**26.60**	**36.60**	**6.10**	**30.50**	--	--	--
XN-6	9.40	.60	8.70	--	--	--	--	--	--	--	--	--
TR-1	**11.30**	3.60	7.70	**13.20**	.20	**13.00**	**15.60**	--	**15.60**	--	--	--
TR-2	8.60	3.70	5.00	--	--	--	--	--	--	--	--	--
TR-3	**19.10**	**5.80**	**13.30**	**29.20**	**15.30**	**13.90**	**33.80**	**17.80**	**16.00**	--	--	--
TR-4	**14.40**	.80	**13.60**	**22.10**	**5.40**	**16.70**	**21.10**	**6.50**	**14.60**	--	--	--
TR-5	8.10	.10	8.00	--	--	--	--	--	--	--	--	--
TR-6	**11.10**	**4.50**	6.60	**26.10**	1.30	**24.80**	**23.40**	--	**23.40**	--	--	--
CK-1	26.70	.60	**26.10**	**30.10**	1.40	**28.70**	**30.30**	--	**30.30**	**29.80**	--	**29.80**
CK-2	20.10	**10.60**	**9.50**	**14.50**	2.90	**11.60**	8.00	--	8.00	--	--	--
CK-3	16.30	1.40	**14.90**	9.30	3.60	5.70	--	--	--	--	--	--
CK-4	**9.10**	1.90	7.20	--	--	--	--	--	--	--	--	--
CK-5	**8.60**	2.60	6.00	--	--	--	--	--	--	--	--	--
CK-6	17.90	.40	**17.60**	**33.10**	0.00	**33.10**	**65.20**	--	**65.20**	**65.20**	--	**65.20**

DC = Danger-Contamination Scale; SES = Socioeconomic Consequences Scale; XN = Xenophobia Scale; TR = Traumatic Stress Scale; CK = Checking Scale; I χ^2^ = overall test of measurement invariance across items; *a* χ^2^ = test of measurement invariance in item discrimination values; *b* χ^2^ = test of measurement invariance in item difficulty parameters. Chi square values from specific measurement invariance tests presented in cells; bold denotes a statistically significant chi square value at p < .05 (the degrees of freedom for the total item tests were 5, 1 for the *a* tests, and 4 for the *b* tests). Significant values here suggest that an item or item parameter may be non-invariant across groups (i.e., significant differences across groups). The initial “All Items” sweep was conducted to identify anchor items and items that may demonstrate non-invariance. This process may over-identify non-invariance however, and so more targeted follow-up tests were conducted using the items and parameters that demonstrated invariance at a previous stage as anchors. The exception was that the presence of non-invariance in the discrimination value suggests that the whole item should be treated as functioning differently across groups, even if there is no evidence for non-invariance in the difficulty parameters (i.e., equal difficulty in the absence of equal discrimination values is not particularly meaningful). In all models the Polish sample was treated as the reference group (factor mean and variance fixed to 0 and 1, respectively) and the Dutch Sample was treated as the focal group (factor and variance freely estimated).

Table 3 includes the Wald test statistics for the total item MI tests (I χ^2^), item discrimination value MI tests (*a* χ^2^), and item difficulty value MI tests (all item difficulty values are tested simultaneously; *b* χ ^2^). Degrees of freedom were either 5 (for the total item tests), 1 (for the discrimination tests), or 4 (for the difficulty tests). Reliable NI was detected for three items of the Danger-Contamination Scale, 0 items of the Socioeconomic Consequences Scale, two items of the Xenophobia Scale, four items of Traumatic Stress Scale, and two items of the Checking Scale. Overall, 11 out of 36 CSS items (31%) demonstrated NI in discrimination and/or difficulty values across countries; on average 35% of the items in a given scale demonstrated some NI.

Item parameter estimates from the final, constrained multi-group graded response models are presented in Table 4; item parameters that were not constrained to equality across groups are presented in bold.

**Table 4:** Results from COVID Stress Scales item response models with parameter constraints across Polish and Dutch samples supported by measurement invariance tests.

	Polish sample					Dutch sample						
	*a*	*b*_1_	*b*_2_	*b*_3_	*b*_4_	*a*	*b*_1_	*b*_2_	*b*_3_	*b*_4_	*d*_MiAssumed_	*d*_MiModeled_
DC-1	3.08	-1.02	-.07	.71	1.68	3.08	-1.02	-.07	.71	1.68	--	--
DC-2	2.87	-.83	-.01	.68	1.78	2.87	-.83	-.01	.68	1.78	--	--
**DC-3**	**1.42**	**-1.90**	**-1.17**	**-.39**	**1.08**	**2.78**	**-.59**	**.17**	**.92**	**1.91**	--	--
**DC-4**	**2.49**	**-1.19**	**-.49**	**.33**	**1.44**	**2.49**	**-1.1**	**-.25**	**.50**	**1.24**	--	--
**DC-5**	**1.30**	**-2.03**	**-1.34**	**-.55**	**1.04**	**2.93**	**-.86**	**-.17**	**.57**	**1.27**	--	--
DC-6	2.39	-.81	-.03	.76	1.79	2.39	-.81	-.03	.76	1.79	--	--
DC-7	4.02	-.83	.14	.76	1.44	4.02	-.83	.14	.76	1.44	--	--
DC-8	3.56	-.72	.15	.77	1.58	3.56	-.72	.15	.77	1.58	--	--
DC-9	3.50	-1.07	-.26	.37	1.23	3.50	-1.07	-.26	.37	1.23	--	--
DC-10	3.19	-.29	.47	1.18	1.96	3.19	-.29	.47	1.18	1.96	--	--
DC-11	2.60	-.11	.70	1.4	2.12	2.60	-.11	.70	1.40	2.12	--	--
DC-12	2.47	.28	1.1	1.79	2.44	2.47	.28	1.1	1.79	2.44	--	--
Scale *d*	--	--	--	--	--	--	--	--	--	--	-.26	-.14
SES-1	3.52	.39	1.04	1.87	2.49	3.52	.39	1.04	1.87	2.49	--	--
SES-2	3.08	.33	1.10	1.98	2.76	3.08	.33	1.10	1.98	2.76	--	--
SES-3	2.65	-.04	.87	1.63	2.43	2.65	-.04	.87	1.63	2.43	--	--
SES-4	4.19	.74	1.32	2.03	2.7	4.19	.74	1.32	2.03	2.70	--	--
SES-5	2.39	.14	.99	1.91	2.82	2.39	.14	.99	1.91	2.82	--	--
SES-6	2.54	.51	1.3	1.87	2.82	2.54	.51	1.30	1.87	2.82	--	--
Scale *d*	--	--	--	--	--	--	--	--	--	--	-.36	-.36
**XN-1**	**3.36**	**.35**	**1.00**	**1.57**	**2.30**	**3.36**	**-.10**	**.68**	**1.38**	**2.19**	--	--
XN-2	5.81	.05	.85	1.42	2.19	5.81	.05	.85	1.42	2.19	--	--
XN-3	8.31	.23	.92	1.44	2.14	8.31	.23	.92	1.44	2.14	--	--
XN-4	2.30	.94	1.65	2.27	2.84	2.30	.94	1.65	2.27	2.84	--	--
**XN-5**	**2.44**	**.72**	**1.34**	**1.92**	**2.59**	**1.75**	**1.39**	**2.17**	**2.74**	**3.84**	--	--
XN-6	2.99	.08	.97	1.60	2.29	2.99	.08	.97	1.60	2.29	--	--
Scale *d*	--	--	--	--	--	--	--	--	--	--	.37	.30
**TR-1**	**3.02**	**.18**	**.67**	**1.34**	**2.43**	**3.02**	**.09**	**.95**	**1.59**	**2.58**	--	--
TR-2	2.60	.71	1.35	2.00	2.92	2.60	.71	1.35	2.00	2.92	--	--
**TR-3**	**3.54**	**-.60**	**.24**	**.91**	**1.94**	**1.99**	**-1.00**	**.00**	**1.37**	**3.46**	--	--
**TR-4**	**4.52**	**-.22**	**.44**	**1.07**	**2.06**	**3.06**	**.07**	**.85**	**1.59**	**2.34**	--	--
TR-5	4.13	.08	.79	1.48	2.22	4.13	.08	.79	1.48	2.22	--	--
**TR-6**	**2.96**	**.22**	**.80**	**1.38**	**2.23**	**2.96**	**.58**	**1.30**	**2.02**	**2.59**	--	--
Scale *d*	--	--	--	--	--	--	--	--	--	--	-.43	-.46
**CK-1**	**1.39**	**-1.99**	**-.80**	**.11**	**1.47**	**1.39**	**-2.07**	**-.98**	**.57**	**2.29**	--	--
CK-2	1.16	-.48	.82	1.96	3.53	1.16	-.48	.82	1.96	3.53	--	--
CK-3	1.62	-.44	.62	1.51	2.79	1.62	-.44	.62	1.51	2.79	--	--
CK-4	1.66	-.68	.18	1.16	2.36	1.66	-.68	.18	1.16	2.36	--	--
CK-5	2.21	.45	1.24	1.99	3.37	2.21	.45	1.24	1.99	3.37	--	--
**CK-6**	**2.48**	**-.91**	**-.11**	**.76**	**1.85**	**2.48**	**.01**	**.70**	**1.49**	**2.43**	--	--
Scale *d*	--	--	--	--	--	--	--	--	--	--	-.97	-.62

DC = Danger-Contamination Scale; SES = Socioeconomic Consequences Scale; XN = Xenophobia Scale; TR = Traumatic Stress Scale; CK = Checking Scale; *a* = item discrimination; *b*_1_…*b*_4_ = item difficulty parameters; *d*_MiAssumed_ = Cohen’s *d* for the scale mean difference between the Polish and Dutch samples with measurement invariance assumed (i.e., all item parameters constrained to equality); *d*_MiModeled_ = Cohen’s *d* for the scale mean difference between the Polish and Dutch samples with measurement invariance modeled (i.e., only invariant item parameters constrained across groups). Items that demonstrated non-invariance are presented in **bold**. Cohen’s *d*s were computed with the Polish sample as the reference group (i.e., positive values denote that the Dutch sample scored higher).

To provide a sense of the practical impact of the NI that was detected, Table 4 also includes Cohen’s *d*s for the mean scale differences across countries while assuming MI (i.e., with all item parameter estimates constrained across groups), and with NI modeled (i.e., with the pattern of parameter constrains presented in Table 4). The items that demonstrated NI tended to be more discriminating, and less difficult, for the Polish sample (average *a* = 2.63; average *b* = .50) compared to the Dutch sample (average *a* = 2.56; average *b* = .97). That is, these items were somewhat more informative, or better at differentiating between individuals, in the Polish sample; the exception here were the items in the Danger-Contamination scale, which were more discriminating for the Dutch sample. Lower values on the latent factors were also generally needed for endorsing higher categories in the Polish sample compared to the Dutch sample. That is, among individuals with similar levels of COVID stress, those in the Polish sample were more likely to endorse a higher response category than those in the Dutch sample.

Although there was evidence of NI in several items, it generally did not appear to have a large effect on group comparisons across countries. Compared to the Dutch sample the Polish sample scored higher on the Danger-Contamination, SES consequences, Traumatic Stress, and Checking scales, and lower on the Xenophobia scale, regardless of if NI was modeled or not (Table 4). NI generally had the effect of slightly exaggerating these group differences. Modeling NI reduced the observed (MI assumed) Cohen’s *d*s by between 19% (from *d* = .37 to *d* = .30 for the Xenophobia Scale) and 46% (from *d* = -.26 to *d* = -.14 for the Danger-Contamination Scale) (there was no NI in the SES Consequences Scale, and NI actually increased the mean difference in the Traumatic Stress Scale by 7%). Overall, when MI was assumed the average Cohen’s *d* was *d* = ± .48, and when NI was modeled the average Cohen’s *d* was *d* = ± .38. Thus, although NI inflated the mean differences, it did not fundamentally change the conclusions regarding the initially observed differences between the two samples.

### Reliability of the CSS-PL

The item parameters presented in Table 4 highlight that in general the CSS scales are psychometrically robust. Item discrimination values were universally large (i.e., greater than *a* = .80; which roughly corresponds to a standardized factor loading of λ = .40), suggesting that all items effectively index the target construct of the specific scales (average *a* across samples and scales = 2.60). Furthermore, item difficulty values were generally spread across a wide range of latent traits.

**Fig 2:**
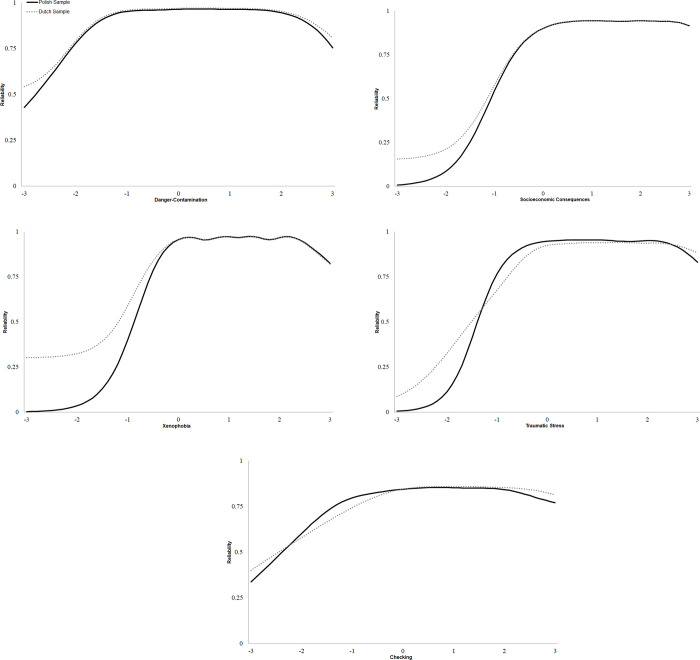
a. Reliability curves for the CSS Danger-Contamination scale for the Polish and Dutch samples. b. Reliability curves for the CSS Socioeconomic Consequences scale for the Polish and Dutch samples. c. Reliability curves for the CSS Xenophobia scale for the Polish and Dutch samples. d. Reliability curves for the CSS Traumatic stress symptoms scale for the Polish and Dutch samples. e. Reliability curves for the CSS Compulsive checking scale for the Polish and Dutch samples. *Note*. Reliability is on the Y axis, latent factor scores are on the X axis in standard deviation units (i.e., 0 corresponds to the factor mean, 1 is one standard deviation above the mean, etc.). Reliability curves are based on the Information Functions from the item response models with parameter constraints supported by DIF tests (see Table 4).

The high discrimination values of the items, and spread of item difficulty values, are jointly captured in Fig 2A–2E, which depicts the scale information functions across samples for each CSS scale (information functions are based on the parameter estimates presented in Table 4).

For ease of interpretability, the original information logits have been converted into rough estimates of reliability [45]. The scales generally provide a reliability of at least *r*_xx_ = .75 from one standard deviation below the mean to up to three standard deviations above the mean). Reliability was generally lowest below one standard deviation below the mean, suggesting that the CSS scales are least effective at measuring (or making finer grained distinctions between) individuals who are not experiencing much COVID-19 stress; on the other hand, these scales appear quite reliable when it comes to measuring individuals experiencing average and higher levels of COVID-19 stress.

The general reliability of the scales across the latent trait were further reflected in the coefficient omega values, which were .94, .91, .93, .93 and .80 for the Danger and Contamination, Socio-Economic Consequences, Xenophobia, Traumatic Stress, and Compulsive Checking, respectively. These scales were all moderately to strongly intercorrelated (see S9 Table), with correlations ranging from *r* = .26 (between Danger and Contamination and Socio-Economic Consequences) to very large (*r* = .69 for Traumatic Stress and Compulsive Checking).

### Test-retest reliability and measurement invariance of the CSS-PL over time

The results from the NI tests across time are presented in S10 Table. This table follows the same format as Table 3. Reliable NI was detected for three items of the Danger-Contamination Scale, zero items of the Socioeconomic Consequences Scale, one item of the Xenophobia Scale, zero items of Traumatic Stress Scale, and two items of the Checking Scale. Overall, 6 out of 36 CSS items (17%) demonstrated NI in discrimination and/or difficulty values across time; on average 15% of the items in a given scale demonstrated some NI.

Item parameter estimates from the final, constrained NI graded response models across time are presented in S11 Table. In general the items that demonstrated NI tended to be more difficult at Time 2 compared to Time 1, especially for the items of the Danger-Contamination scale. Overall though only a small number of items demonstrated NI, and the NI that was identified appeared to be relatively modest in size. Differences between parameter estimates were small, and mean differences over time were largely unaffected (SX). Scores on all 5 scales decreased over time—Cohen’s *d*s from -.03 (Socioeconomic Consequences) to -.49 (Compulsive Checking)—and only the change over time in the Danger-Contamination scale was affected (very modestly) by NI (the *d* decreased in magnitude by .03).

The correlations for the CSS-PL scales from Time 1 to Time 2 were typically large in magnitude. Specifically, the correlations for the scales across time were: .73 (Danger and Contamination), .40 (Socioeconomic Consequences), .57 (Xenophobia), .70 (Traumatic Stress), and.64, (Compulsive Checking).

### Convergent, discriminant and criterion associations of the CSS-PL

Correlations between the CSS-PL scales and the various criterion measures are presented in Table 5.

**Table 5:** Correlations between the Polish COVID-Stress Scales (CSS-PL) and other psychological constructs.

	COVID-Stress Scales-PL				
Variables	COVID danger and contamination	COVID socioeconomic consequences	COVID xenophobia	COVID traumatic stress symptoms	COVID compulsive checking
**Tests of convergent validity**					
Fear of COVID-19^a^	.77***	.32***	.54***	.83***	.67***
Health anxiety^b^	.60***	.17**	.39***	.55***	.37***
OC checking^b^	.31***	.15*	.24***	.40***	.28***
OC washing^b^	.56***	.21***	.35***	.49***	.29***
**Tests of discriminant validity**					
Anxiety^b^	.44***	.17**	.27***	.53***	.20***
Depression^b^	.35***	.17**	.34***	.36***	.16**
Social Desirability^b^	.05	.07	.03	.00	.08
Xenophobia^b^	-.05	.10	.25***	.00	.04
Sensation seeking^b^	-.22***	.09	-.06	-.09	-.10
Tests of concurrent and predictive criterion validity					
Intent to have a COVID-19 vaccine^c^	.52***	-.06	.17***	.31***	.24***
	.45***	-.16*	.10	.23**	.17*

*Note*. ^a^ Sample at T1 (N = 556). ^b^ Sample at T2 (*n* = 264). ^c^ The top rows include the correlations between intent to have a COVID-19 vaccine at T1 and CSS-PL at T1 in a subsample of 362 participants who did not have a COVID-vaccine at T1. The bottom rows include the correlations between intent to have a COVID-19 vaccine at T2 and CSS-PL at T1 determined in a subsample of 165 individuals who were not vaccinated at T2.

*** *p* < .001.

** *p* < .01.

* p < .05.

The associations between the five CSS-PL scales and COVID-19-related fear (measured by the Fear of COVID-19 Scale; FCV-19S), health anxiety-related traits (measured by the Short Health Anxiety Inventory; SHAI), and obsessive-compulsive washing and checking (measured by the Obsessive Compulsive Inventory-Revised; OCI-R) were all positive and strong (Table 5), confirming the convergent validity of the CSS-PL.

The divergent associations between the CSS-PL scales and general anxiety and depression (measured by the Patient Health Questionnaire-4; PHQ-4), xenophobia (measured by the Political Beliefs Questionnaire; PBQ) and social desirability (measured by the Social Desirability Scale; SDS-17) were all consistent with the pattern of associations expected based on the results of the original CSS development study by Taylor and colleagues [8] (see S12 and S13 Tables for tests of the differences between correlations). All the CSS-PL sales were positively correlated with anxiety and depression, and these correlations ranged from small to medium to large and very large in magnitude. In turn, the CSS-PL Xenophobia Scale was the only CSS-PL scale that was correlated with xenophobia. None of the CSS-PL scales were correlated with the SDS-17 assessing social desirability. Finally, an additional discriminant variable—sensation seeking as measured by the Impulsive Behavior Scale Short Version (SUPPS-P)—only demonstrated a moderate, negative correlation with the COVID Danger-Contamination scale.

With respect to the concurrent criterion validity, correlations between most of the CSS-PL scales at T1 (the exception was the COVID Socioeconomic Consequences scale) and intent to receive a COVID-vaccine at T1 were small-to moderate to strong. With respect to the predictive criterion validity, correlations between the CSS-PL at T1 and intent to receive a COVID-vaccine after four weeks at T2 were significant and positive (the exception was the non-significant correlation with the COVID Xenophobia scale), and their magnitude ranged from small to strong.

## Discussion

The present study described the initial development and evaluation of the Polish-language version of the COVID Stress Scales (CSS-PL) and in so doing also managed to extend prior psychometric vetting of the CSS more broadly [8].

### Psychometric functioning of CSS-PL across countries

Consistent with the original CSS [8], as well as other language versions of the CSS (e.g., Persian and Arabic) [11,43, 44] a 5 factor structure of the CSS-PL appeared reasonable. This further suggests that across cultural context the items of the CSS tend to cohere into a similar handful of factors (though in certain samples more or less factors may also be defensible), and that these factors are moderately to strongly interrelated, providing evidence for the COVID Stress Syndrome across diverse nations.

Our evaluation of measurement invariance also helped to highlight areas of potential similarity and difference when measuring COVID stress across distinct cultural contexts. Overall, slightly less than a third of the items demonstrated some non-invariance (NI) across the Polish and Dutch samples. However, this NI did not seem to be particularly large or impactful in magnitude. Still, results illustrate the importance of at least considering MI when examining and comparing COVID stress across cultural contexts (including developing different language versions), and accounting for it in subsequent analyses when necessary (again, by either revising items, dropping them, or incorporating NI into the measurement model). Tests of NI can also help identify potentially important cultural differences across contexts (i.e., NI may be substantively meaningful), and be used to detect when certain items of the CSS have not translated well across contexts [46, 47].

Despite the minor differences in psychometric functioning, both the Polish and Dutch versions of the CSS appeared very reliable. Item discrimination values were universally large (i.e., *a* > .80), and reliability estimates were greater than *r*_xx_ = .75 across the range of all scales, especially in the higher range. That is, the CSS well-suited to measuring individuals with slightly-below average to well-above average levels of COVID stress; conversely, the scales are least reliable for those individuals experience little to no COVID stress. This somewhat asymmetric distribution of the reliability curves is consistent with the overall intention of the scale, however. That is, the goal is the CSS is primarily to identify and rank-order individuals experiencing moderate to large degrees of COVID stress. Relatedly, the pattern of correlations between the CSS-PL scales (ranging from medium to very large) was consistent with the pattern of correlations obtained by Taylor and colleagues [8] and Khosravani and colleagues [11] in regard to the Persian CSS. This, along with our other psychometric results, provides further support for the notion that symptoms measured in the CSS (here CSS-PL) constitute a coherent COVID Stress Syndrome that appears to be a cross-cultural experience [8, 11].

### Psychometric functioning of CSS-PL across time

Measurement invariance is critical to consider both when comparing groups, and when analyzing change over time. Overall the CSS-PL appeared largely invariant over time. Few items demonstrated NI, and for those that did the differences in the parameter estimates across time, and the impact on estimates of mean change, were negligible. Admittedly four weeks is not a large gap, and so major psychometric changes might not be expected. However, in the context of the COVID-19 pandemic there has a been a steady, rapid stream of developments regarding the threat level, appropriate safety behaviors, vaccine availability, that give rise for hopes for effective treatment options of the COVID-19 [14]. The dynamically changing nature of pathogenesis, diagnosis, prognosis, and treatment options in the COVID-19 pandemic [14] may, therefore, encourage researchers to use shorter intervals between assessments in future studies to capture how the rapidly changing nature of the COVID-19 pandemic may affect stress and anxiety related to the COVID-19.

Although the measurement parameters were generally stable over time, the scale means were lower at T2. That is, the psychometric properties of the CSS might not change much in response to pandemic developments—which is preferable all else being equal—but the scales themselves still detected a considerable amount of non-trivial change over a relatively short period. Rank-order stability was generally high though, which was consistent with what was observed in the recent examination of the Persian CSS [11]. It is important to note though that the CSS was created as a measure of symptoms not as a measure of traits [7] so it is likely that CSS scores may fluctuate in response to increasing or decreasing rates of infections [6]. Taken together results suggest that on average COVID stress decreased over the assessment period, however, individuals experiencing more COVID stress than others at T1 were more likely to score high on COVID stress at T2 as well.

### Correlates of the CSS-PL

Convergent associations between the five CSS-PL subscales and the Fear of COVID-19 Scale (FCV-19S) assessing fear of COVID-19 [21] were moderate to strong in size. Positive correlations between the FCV-19S and CSS have been also demonstrated in other studies aiming at developing non-English versions of the CSS-PL (e.g., the Persian and Arabic versions of the CCS [11, 44]. This pattern of associations between the CSS and FCV-19S also suggest that the FCV-19S appears to capture two constructs measured by the CSS, i.e., the danger and contamination and the traumatic symptoms and checking constructs [48]. The CSS-PL scales also demonstrated moderate to large associations with measures of anxiety-related traits and obsessive-compulsive symptoms. This is consistent with past findings on the CSS [8, 11] and further support the convergent validity of the CSS-PL.

Discriminant associations for the CSS-PL were also consistent with past findings [8, 11]. That is, the CSS-PL was not significantly correlated with measures of social desirability or impulsivity, the CSS-PL Xenophobia scale was more strongly correlated with the General Xenophobia Scale than the other scales, while the other CSS scales were more strongly correlated with anxiety and depression than the Xenophobia scale. Relatedly, most of the CSS scales were more strongly correlated with current anxiety than depression, however, this difference was only statistically significant for the Traumatic Stress scale. The lack of a consistent difference between associations with anxiety and depressive symptoms may be related to the timing of data collection. Data on the CSS was originally collected at the beginning of pandemic [8] while the current data was collected roughly a year after the outbreak of the pandemic. It could be the case that fear and anxiety surrounding the outbreak was more distinct from depression in the early days of the outbreak compared to after one year of dealing with the outbreaks consequences.

Finally, we assessed both concurrent and predictive associations between the CSS-PL and intent to receive a COVID-19 vaccine. We found moderate to strong correlations between three CSS-PL scales at T1 (Danger and Contamination, Traumatic Stress Symptoms, Compulsive Checking) and intent to receive a COVID vaccine at T1. That is, higher levels of these fears were associated with greater intent to be vaccinated. Further, the Danger and Contamination, Socioeconomic Consequences, Traumatic Stress Symptoms and Checking scales assessed at T1 were associated with intent to receive a COVID-19 vaccine after 1-month interval. Thus, the intent to be vaccinated at T2 was stronger among those experiencing more COVID stress at T1. These results are consistent with prior research showing that higher anxiety is related to a greater likelihood of protective behaviors in past pandemics [26], and greater motivation for social distancing in the case of the current COVID-19 pandemic [27]. Thus, our findings help to extend prior work concerning protective hygiene behaviors by demonstrating how the CSS can predict important, COVID-related hygiene behaviors such as vaccination, while highlighting the role of psychological factors in helping to understand the intent to receive a COVID-19 vaccine [23–25].

## Limitations

Despite a number of strengths, the present work also has limitations that need to be considered when interpreting the findings. First, although the pandemic in Poland as in other countries is likely to be a source of extreme stress [9, 10], it is also probable that state of the pandemic vary across countries and as a consequence, the levels and experiences of stress and fears related to COVID-19 also may differ across countries.

Second, the sample utilized in the current study may not be representative of the entire Polish population in several aspects. The recruitment of participants via Facebook did not reach participants who do not use this website, as well as those individuals who do not use the Internet (as may be the case for older individuals). Indeed, although our investigation included participants across a wide age range (from 18 to 85 years), participants 60 years and older represented only 17.30% of the total sample (*n* = 96). This age group is particularly salient given the mortality rates of COVID-19, and potentially more COVID-19 fear, among older individuals [48]. Further, our sample was over 60% female, and there is evidence for gender differences in regard to attitudes and behaviors towards COVID-19 (e.g., women were more likely to perceive COVID-19 as a very serious health problem and support/comply with restrictive public policies in response to COVID-19 [49].

Third, data for this study was collected between February and April, 2021, one year since the COVID-19 pandemic outbreak, whereas the original study on the CSS was conducted in parallel with the beginning of the pandemic. Moreover, the current investigation was performed after the development and distribution of several highly-effective COVID-19 vaccines. These circumstances are likely to influence the stress and fears experienced by our study participants at the time of the assessment and could affect certain aspects of the findings (e.g., item difficulty estimates; some fears may be more difficult to endorse given the availability of vaccines in certain countries). Further, in the current investigation we used a 4-week between assessments, which is relatively short. Future research would benefit from employing both much shorter and longer intervals to better understand how the measurement and manifestation of COVID-19 stress changes over different stretches of time in the context of a rapidly changing pandemic [14].

Fourth, although we asked participants about type of employment they were engaged in we did not ask about their type of employment. Essential workers for example, experience a greater risk of being potentially infected, which likely has consequences for COVID-19 stress. Participants were also not asked about whether they take care of a child/children, older parents, or other dependents, which also might contribute to various facets of stress related to COVID-19, especially given how prominent concerns for the health of loved ones have been in the current pandemic [6]. Future research would benefit from inclusion of these two additional variables.

Finally, the high attrition rate (52.50%) between the first and second assessment is a major limitation. However, although we detected some differences in baseline demographic variables across time, these differences were very and small in magnitude and the possibility of bias should not be serious. Relatedly, as noted above, 4-weeks may be too short of a time interval to expect NI to become a major concern, which leaves the broader implications of the over-time tests of invariance ambiguous (i.e., to what extent are the measurement properties of the CSS stable over longer periods). However, given the rapidly changing situation regarding the pandemic, short-term measurement invariance is still a potentially important issue worth examining.

## Conclusion

The current study demonstrates that the Polish version of the CSS (CSS-PL)—like the original CSS—has a defensible 5-factor structure and robust psychometric properties in terms of reliability and validity. Furthermore, the CSS may be invariant across countries and time, at least over and brief time intervals. The CSS-PL thus appears to be a promising multidimensional instrument for assessing COVID-related stress in the Polish population. The current findings based on the Polish CSS also extend past work on general the psychometric properties of the CSS, which can be useful for researchers across contexts who are interested in assessing the COVID-19 Stress Syndrome.

## Supporting information

S1 File (DOCX)

S2 File (DOCX)

S1 Table Steps in the translation procedure of the original COVID-19 Stress Scales (CSS) into the Polish-language. The *COVID-19 Stress Scales (CSS)* (Taylor et al., 2020a) were translated following the recommendations of the “ISPOR Task Force for Translation and Cultural Adaptation” (Wild et al., 2005) using a 10-step procedure for translation.
(DOCX)

S2 Table Descriptive and cross-language comparison of the original CSS item scores and the CSS-PL_EX in the study 1 with bilingual participants. *M* = mean; *SD* = standard deviation; *t* = *t*-test; *p* = *p*-value; *r* = Pearson’s correlation between the Polish and the corresponding English items. * p < .05. ** *p* < .01.
(DOCX)

S3 Table Descriptive, internal consistency reliability, and cross-language comparison of the polish experimental version of the CSS (CSS-PL_EX) and the original CSS scales scores in study 1. *M* = mean; *SD* = standard deviation; avg. *r*_it_ = average item-total correlation; *t* = *t*-test; *r* = Pearson’s correlation between the subscales’ scores of the CSS-PL_EX and the original CSS. ** *p* < .001, two—tailed. * *p* < .05 two—tailed.
(DOCX)

S4 Table Factor loadings from 1 factor exploratory structural equation model. CSS = COVID Stress Scale Item; F1 = Factor 1. Models fit using weighted least squares with mean and variance adjustment (WLSMV) estimation. Factor loadings greater than λ = ±.40 presented in bold.
(DOCX)

S5 Table Factor loadings from 4 factor exploratory structural equation model. CSS = COVID Stress Scale Item; F1…F4 = Factors 1 through 4. Models fit using weighted least squares with mean and variance adjustment (WLSMV) estimation and targeted oblique rotation. The rotation targets for items not associated with a factor were set to 0. Factor loadings greater than λ = ±.40 presented in **bold.** Factor correlations ranged from *r* = .20 to .64 (average *r* = .34) in the Polish sample, and from *r* = .26 to .56 (average *r* = .37) in the Dutch sample.
(DOCX)

S6 Table Factor loadings from 5 factor exploratory structural equation model. CSS = COVID Stress Scale Item; F1…F5 = Factors 1 through 5. Models fit using weighted least squares with mean and variance adjustment (WLSMV) estimation and targeted oblique rotation. The rotation targets for items not associated with a factor were set to 0. Factor loadings greater than λ = ±.40 presented in bold. Factor correlations ranged from *r* = .05 to .55 (average r = .28) in the Polish sample, and from r = .04 to .49 (average *r* = .31) in the Dutch sample.
(DOCX)

S7 Table Factor loadings from 6 factor exploratory structural equation model. CSS = COVID Stress Scale Item; F1…F6 = Factors 1 through 6. Models fit using weighted least squares with mean and variance adjustment (WLSMV) estimation and targeted oblique rotation. The rotation targets for items not associated with a factor were set to 0. Factor loadings greater than λ = ±.40 presented in **bold.** Factor correlations ranged from *r* = .05 to .55 (average *r* = .28) in the Polish sample, and from *r* = .04 to .49 (average *r* = .31) in the Dutch sample.
(DOCX)

S8 Table Factor loadings from 5 factor correlated factors model. CSS = COVID Stress Scale Item; F1…F5 = Factors 1 through 5. Models fit using weighted least squares with mean and variance adjustment (WLSMV) estimation. The rotation targets for items not associated with a factor were set to 0. Factor correlations ranged from *r* = .31 to .82 (average *r* = .54) in the Polish sample, and from *r* = .43 to .75 (average *r* = .57) in the Dutch sample. Model fit for the Polish sample was: χ^2^ = 3447.67, df = 584, *p* < .01; RMSEA = .094; SRMR = .08; CFI = .919; TLI = .912. Model fit for the Dutch sample was: χ^2^ = 1720.01, df = 585, *p* < .01; RMSEA = .071; SRMR = .076; CFI = .937; TLI = .933. Dutch sample has one extra degree of freedom because the factor loading for CSS-10 was fixed to .98 to avoid convergence with negative residual variances.
(DOCX)

S9 Table Reliability of the Polish COVID-Stress Scales (CSS-PL) and correlations among the CSS-PL Scales. *** *p* < .001.
(DOCX)

S10 Table Results from COVID Stress Scale measurement invariance tests across times 1 and 2. DC = Danger-Contamination Scale; SES = Socioeconomic Consequences Scale; XN = Xenophobia Scale; TR = Traumatic Stress Scale; CK = Checking Scale; I χ^2^ = overall test of measurement invariance across items; *a* χ^2^ = test of measurement invariance in item discrimination values; *b* χ^2^ = test of measurement invariance in item difficulty parameters. Chi square values from specific measurement invariance tests presented in cells; bold denotes a statistically significant chi square value at p < .05. Significant values here suggest that an item or item parameter may be non-invariant across time (i.e., significant differences across time).The initial “All Items” sweep was conducted to identify anchor items and items that may demonstrate non-invariance. This process may over-identify non-invariance however, and so more targeted follow-up tests were conducted using the items and parameters that demonstrated invariance at a previous stage as anchors. The exception was that the presence of non-invariance in the discrimination value suggests that the whole item should be treated as functioning differently across time, even if there is no evidence for non-invariance in the difficulty parameters (i.e., equal difficulty in the absence of equal discrimination values is not particularly meaningful). In all models Time 1 was treated as the reference group (factor mean and variance fixed to 0 and 1, respectively) and Time 2 was treated as the focal group (factor and variance freely estimated).
(DOCX)

S11 Table Results from COVID Stress Scale item response models with parameter constraints across times 1 and 2 supported by DIF tests. DC = Danger-Contamination Scale; SES = Socioeconomic Consequences Scale; XN = Xenophobia Scale; TR = Traumatic Stress Scale; CK = Checking Scale; *a* = item discrimination; *b*_1_…*b*_4_ = item difficulty parameters; *d*_MiAssumed_ = Cohen’s *d* for the scale mean difference across time with measurement invariance assumed (i.e., all item parameters constrained to equality); *d*_MiModeled_ = Cohen’s *d* for the scale mean difference across time with measurement invariance modeled (i.e., only invariant item parameters constrained across time). Items that demonstrated non-invariance are presented in **bold**. Cohen’s *d*s were computed with Time 1 as the reference group (i.e., positive values denote that scores were higher at Time 2).
(DOCX)

S12 Table Tests of discriminant validity: Comparison of the CSS-PL correlations with current anxiety and depression. Sample of 264 participants at Time 2. Correlations ≥ .30 in bold.
(DOCX)

S13 Table Tests of discriminant validity: Comparison of the mean correlations for distress measures versus correlation with general xenophobia. General distress = correlations of the CSS-PL with the measures of general anxiety, depression, and trait measures of health anxiety and OC symptoms at Time 2. Sample of 264 participants at Time 2. Correlations ≥ .30 in bold.
(DOCX)
