# Supplementary material for: The Polish COVID Stress Scales: Considerations of psychometric functioning, measurement invariance, and validity
Source: PLoS One. 2021 Dec 1;16(12):e0260459. doi: 10.1371/journal.pone.0260459 (PMC8635383; doi:10.1371/journal.pone.0260459)
Supplement: S1 File — (DOCX) [file pone.0260459.s001.docx]

**Polish translation of the CSS and its equivalence with the original CSS**

With the permission of the original authors of the CSS (Taylor et al., 2020a), the original CSS was translated into the Polish language following the recommendations of the “ISPOR Task Force for Translation and Cultural Adaptation” (Wild et al., 2005) by the employment of a 10-step procedure for translation (Table S1 in online supplemental materials). The Polish translations of the CSS can be taken from the online supplemental materials.

The experimental Polish version of the CSS (CSS-PL_EX) was employed in a sample of 38 bilingual university students (Study 1) from the English Faculty at Adam Mickiewicz University through the advertisement sent via university e-mails. Data were collected between February 1 and March 15, 2021. The recruitment resulted in a full data set of 38 respondents who participated in two stages of the study (30 females, 6 males, 2 participants indicated other gender) who were aged from 19 to 32 (*M* = 21.34, *SD* = 2.67). In the first stage, participants were asked complete the original English version of the CSS, whereas after a one-month interval participants were asked to complete the CSS-PL_EX.

In Study 1, the analyses demonstrated significant differences between the pairs of the Polish and English items no. 2, 4, 7, 8, 9, 13, 15, 19, 25, 26, 29, 30, and 33 (Table S2 in online supplemental materials). Specifically, the mean values for the enumerated above items were higher for the English CSS in comparison to the CSS-PL_EX. Further, the correlations between corresponding CSS items in English and Polish languages were significant and positive with the exception of the pair of items no. 7 (*r* = .28, *p* = .095). The correlations between remaining 35 items were significant and were considered as ranging from medium (from *r* = .35 for a pair of items no. 12) to large and very large (*r* = .78 for a pair of items no. 1) (Table S2 in online supplemental materials).

Next, we determined the mean-level differences between the Polish and English CSS five scales (Table S3 in the supplemental online materials). The significant mean-level differences emerged between the Polish and English COVID traumatic stress symptoms subscales, *t*(37) = 2.62, *p* =. 013, Cohen’s *d* = 0.34, on which bilingual participants scored higher in the English version than in the Polish version. Finally, the five original CSS scales were significantly, strongly and positively correlated with the five CSS-PL_EX scales and estimates of internal consistency reliability were high and satisfactory in both the English and Polish versions of the CSS (Table S3 in online supplemental materials).

Based on the results obtained in the Study 1, we employed an independent translator to review the items in regard to which we determined the lack of significant correlation in Polish and English language versions (item no. 7) as well as those items in regard to which we determined existence of the mean-level differences (items no. 2, 4, 7, 8, 9, 13, 15, 19, 25, 26, 29, 30, 33). Based on this procedure, we revised the problematic items and in the pilot Study 2 we utilized an improved, revised version of the Polish CSS termed a pre-final CSS-PL (CSS-PL_PF).

In the pilot Study 2 testing the Polish pre-final translation of the CSS, the CSS-PL_PF was administered along with a 6-item set of questions concerning basic demographic data, i.e., age, gender, place of residence, education, employment, and whether a respondent was infected by the coronavirus. Data were collected between March 17 and March 26, 2021 in an online survey. The sample was recruited using the snowball method through the advertisement sent via e-mails. Initially, 128 respondents began the online survey, however two respondents who completed the survey twice and one person who did not provide informed consent were excluded from further analyses. Therefore, the final sample involved 125 participants who were aged 19 - 73 (*M* = 30.71, *SD* = 13.69). Women represented 68.80% of the sample (*n* = 86), men represented 29.60% (*n* = 37), and 1.60% (*n* =2) respondents indicated other gender. Most of the participants (*n* = 43; 34.40%) lived in a city with more than 500,000 inhabitants. Students represented 49.60% (*n* = 62), 48 participants (38.40%) were degree holders, 13 respondents (10.40%) completed secondary education and 2 respondents (1.60%) completed vocational education. With respect to the employment, most of respondents (*n* = 42; 33.60%) were employed based on the contract of employment. Among respondents, 17 participants (13.60%) indicated that they were infected by the coronavirus.

To examine the factorial structure of the CSS-PL_PF, we performed exploratory factor analysis using Principal axis factoring (PAF) which is a least-squares estimation of the common factor model (de Winter & Dodou, 2012). Analogically, as in the original study by Taylor et al. (2020a), we utilized oblique (Oblimin) rotation and parallel analysis to determine the number of factors to retain. The analysis was run using the Jamovi version 1.6.15 software. As a supportive method to retain the factors, we used a web-based parallel analysis engine (Patil et al., 2017). Specifically, this application calculates eigenvalues from randomly generated correlation matrices that can be subsequently compared with eigenvalues extracted from the researcher's dataset, and the number of factors to retain are the number of eigenvalues that were generated from the researcher’s dataset that were larger than the corresponding random eigenvalues (Horn, 1965). In our parallel analysis we used the default (and recommended) 100 values for number of random correlation matrices and 95 percentile of eigenvalues (see Patil et al., 2017).

Initially we examined factorability of the variables, before conducting the parallel and the factor analyses. The Kaiser-Meyer-Olkin test of sampling adequacy (.79) indicated that the data were middling (Kaiser & Rice, 1974), and the Bartlett’s test (χ² = 2511, *df* = 630, *p* < .001) indicated that the data were factorable. Parallel analysis provided the results with the following eigenvalues: 7.76, 3.22, 2.86, 1.48, 0.97, and 0.78. The 36 items and these six eigenvalues (Table S4 in online supplemental materials) accounted for 53.30% of the cumulative variance in the model. Further, the CSS-PL_PF items no. 1 and 2 had a salient loading (> .30) on both factors, i.e., on COVID contamination and COVID danger, whereas in the original study, these two items had a salient loading only on one factor, i.e., on COVID-related danger; the CSS-PL_PF item no. 17 did not have a salient loading on none of factors.

In the next step, we compared the eigenvalues obtained in the analysis with the parallel average random eigenvalues calculated based on the parameters provided in the web-based parallel analysis engine (Patil et al., 2017). These eigenvalues were as follows: 1.53, 1.34, 1.22, 1.11, 1.01 and 0.92. Examination of the eigenvalues obtained in the current analysis with the parallel average random eigenvalues indicated that only the first four eigenvalues were greater than those generated from randomly generated correlation matrices. This examination suggested therefore four factors to retain. Hence, we fixed extraction to four factors for which we retained the same names in the original study by Taylor and colleagues (2020a), i.e., (1) COVID danger and contamination (12 items, ω = .88), (2) COVID fears about economic consequences (six items, ω = .87), (3) COVID xenophobia (six items, ω = .84), (4) COVID contamination fears (six items, ω = .89), and (5) COVID traumatic stress symptoms and compulsive checking and reassurance seeking (12 items, ω = .86).

**References (not included in the article)**

de Winter, J. C.F., & Dodou, D. (2012). Factor recovery by principal axis factoring and maximum likelihood factor analysis as a function of factor pattern and sample size. *Journal of Applied Statistics, 39*(4), 695-710. doi: [10.1080/02664763.2011.610445](https://doi.org/10.1080/02664763.2011.610445)

Horn, J. L. (1965). A rationale and test for the number of factors in factor analysis. *Psychometrika, 30*(2), 179–185. https://doi.org/10.1007/BF02289447

Kaiser, H. F., & Rice, J. (1974). Little Jiffy, Mark IV. *Educational and Psychological Measurement, 34*(1), 111–117. <https://doi.org/10.1177/001316447403400115>

Patil, V. H., Surendra, N., S., Sanjay, M., & Donavan, D. T. (2017). Parallel analysis engine to aid in determining number of factors to retain using R [Computer software], available from https://analytics.gonzaga.edu/parallelengine/
