# Supplementary material for: The Polish COVID Stress Scales: Considerations of psychometric functioning, measurement invariance, and validity
Source: PLoS One. 2021 Dec 1;16(12):e0260459. doi: 10.1371/journal.pone.0260459 (PMC8635383; doi:10.1371/journal.pone.0260459)
Supplement: S2 File — (DOCX) [file pone.0260459.s002.docx]

**Direct Translation 1.**

Następujące stwierdzenia dotyczą różnych obaw, jakich mogłe/aś doświadczyć w ciągu ostatnich siedmiu dni. W poniższych stwierdzeniach COVID-19 określany jest słowem “wirus”.

Obawiam się złapania wirusa

Obawiam się, że podstawowe zabiegi higieniczne (np. mycie rąk) nie wystarczają, aby uchronić się przed wirusem

Obawiam się, że nasz system opieki zdrowotnej nie jest w stanie ochronić mnie przed wirusem

Obawiam się, że nie potrafię ochronić mojej rodziny przed wirusem

Obawiam się, że nasz system opieki zdrowotnej nie będzie w stanie ochronić moich bliskich

Obawiam się, że dystans społeczny nie wystarczy mi, aby uchronić się przed wirusem

Obawiam się, że w sklepach zabraknie jedzenia

Obawiam się, że w sklepach zabraknie środków na przeziębienie lub grypę

Obawiam się, że w aptekach zabraknie leków na receptę

Obawiam się, że w sklepach zabraknie wody

Obawiam się, że w sklepach zabraknie produktów myjących lub dezynfekujących

Obawiam się, że sklepy zostaną zamknięte

Obawiam się, że obcokrajowcy roznoszą wirusa w moim kraju

Gdybym spotkał/a osobę z innego kraju, obawiał(a)bym się, że mogą być zakażeni wirusem

Obawiam się kontaktów z obcokrajowcami, ponieważ mogą być zakażeni wirusem

Obawiam się, że obcokrajowcy roznoszą wirusa, ponieważ nie zachowują takiej higieny jak my

Gdybym poszedł/ła do restauracji serwującej dania z innych krajów, obawiał(a)bym się złapania wirusa

Gdybym znajdował/a się w windzie z grupą obcokrajowców, obawiał(a)bym się, że są oni zakażeni wirusem

Obawiam się, że ludzie wokół mnie zarażą mnie wirusem

Obawiam się, że jeśli dotknąłbym/ęłabym czegoś w miejscu publicznym (np. poręczy, klamki), złapał(a)bym wirusa

Obawiam się, że gdyby ktoś zakaszlał lub kichnął blisko mnie, złapał(a)bym wirusa

Obawiam się, że mógłbym/mogłabym złapać wirusa przez dotykanie pieniędzy lub używanie terminala

Obawiam się odbierania reszty podczas transakcji gotówkowych

Obawiam się, że moje przesyłki pocztowe zostały zainfekowane przez osoby pracujące na poczcie

W poniższych stwierdzeniach COVID-19 określany jest słowem “wirus”. Przeczytaj każde stwierdzenie i wskaż, jak często dany problem występował u ciebie w ciągu ostatnich siedmiu dni.

Miałem/am problemy ze snem, ponieważ martwiłem/am się wirusem

Miałem/am złe sny związane z wirusem

Myślałem/am o wirusie, kiedy nie miałem/am takiego zamiaru

W mojej głowie pojawiały się niepokojące wyobrażenia dotyczące wirusa wbrew mojej woli

Miałem/am problemy z koncentracją, ponieważ cały czas myślałem/am o wirusie

Czynniki przypominające o wirusie sprawiały, że występowały u mnie fizyczne reakcje, takie jak pocenie czy przyspieszone bicie serca

Poniższe punkty dotyczą zachowań związanych ze sprawdzaniem. Jak często sprawdzałeś/aś poniższe rzeczy z powodu obaw związanych z COVID-19 w ciągu ostatnich siedmiu dni?

Posty w mediach społecznościowych dotyczące COVID-19

Filmy na YouTubie dotyczące COVID-19

Poszukiwanie wsparcia ze strony znajomych i rodziny w sprawie COVID-19

Sprawdzanie własnego ciała w poszukiwaniu śladów infekcji (np. mierzenie temperatury)

Proszenie o poradę specjalistów związanych z ochroną zdrowia (np. lekarzy lub farmaceutów) w sprawie COVID-19

Poszukiwanie informacji dotyczących leczenia COVID-19 w Internecie

**Direct Translation 2.**

Poniższe pytania dotyczą różnych rodzajów obaw, których mógł (mogła) Pan(/-i) doświadczyć w ciągu ostatnich siedmiu dni. W poniższych zdaniach słowo „wirus” odnosi się do COVID-19.

Obawiam się, że złapię wirusa.

Obawiam się, że podstawowa higiena (np. mycie rąk) nie wystarczy, aby uchronić mnie przed wirusem.

Obawiam się, że nasz system opieki zdrowotnej nie jest w stanie uchronić mnie przed wirusem.

Obawiam się, że nie mogę uchronić mojej rodziny przed wirusem.

Obawiam się, że nasz system opieki zdrowotnej nie będzie w stanie ochronić moich najbliższych.

Obawiam się, że dystans społeczny nie wystarczy, aby uchronić mnie przed wirusem.

Obawiam się, że w sklepach spożywczych może zabraknąć jedzenia.

Obawiam się, że w sklepach spożywczych nie będzie leków na przeziębienie i grypę.

Obawiam się, że w aptekach zabraknie leków na receptę.

Obawiam się, że w sklepach spożywczych zabraknie wody.

Obawiam się, że w sklepach zabraknie środków czyszczących lub dezynfekujących.

Obawiam się, że sklepy spożywcze zostaną zamknięte.

Obawiam się, że cudzoziemcy rozprzestrzeniają wirusa w moim kraju.

Gdybym spotkał obcokrajowca obawiałbym/obawiałabym się, że może być zarażony wirusem.

Obawiam się kontaktu z obcokrajowcami, ponieważ mogą być zarażeni wirusem.

Obawiam się, że obcokrajowcy roznoszą wirusa, ponieważ nie dbają o higienę tak jak my.

Gdybym poszedł/poszła do restauracji specjalizującej się w kuchni orientalnej, martwiłbym/martwiłabym się, że złapię wirusa.

Gdybym był(/-a) w windzie z grupą obcokrajowców, martwiłbym/martwiłabym się, że są zarażeni wirusem.

Obawiam się, że ludzie w moim otoczeniu zarażą mnie wirusem.

Obawiam się, że jakbym dotknął(/-ęła) czegoś w miejscy publicznym (np. poręczy, klamki) to zaraziłbym/zaraziłabym się wirusem.

Obawiam się, że gdyby ktoś obok mnie kaszlnął lub kichnął, to zaraziłbym/zaraziłabym się wirusem.

Obawiam się, że mogę zarazić się wirusem podczas płatności gotówką lub kartą.

Obawiam się przyjmowania reszty w gotówce.

Obawiam się, że moje listy lub paczki zostały zakażone przez pracowników poczty.

W poniższych zdaniach słowo „wirus” odnosi się do COVID-19. Przeczytaj każde zdanie i wskaż, czy i jak często dany problem występował u Pana(/-i) w ciągu ostatnich siedmiu dni.

Miałem(/-am) problemy ze snem, ponieważ martwiłem(/-am) się wirusem.

Miałem(/-am) koszmary o wirusie.

Dręczyły mnie myśli o wirusie.

Wbrew mojej woli pojawiały się w mojej głowie niepokojące obrazy związane z wirusem.

Miałem(/-am) problemy z koncentracją, ponieważ ciągle myślałem(/-am) o wirusie.

Wzmianki o wirusie powodowały u mnie reakcje fizyczne, takie jak pocenie się lub przyspieszone bicie serca.

Poniższe punkty mają na celu zbadanie zachowania. Jak często w ciągu ostatnich siedmiu dni ze względu na obawy dotyczące COVID-19 szukał(/-a) Pan(/-i):

informacji w mediach społecznościowych dotyczących COVID-19,

filmów na YouTube o COVID-19,

podniesienia na duchu u znajomych lub rodziny w związku z COVID-19,

u siebie oznak infekcji (np. mierzenie temperatury),

porady u pracowników służby zdrowia (np. lekarzy lub farmaceutów) w związku z COVID-19,

informacji o metodach leczenia COVID-19 w Internecie.

**Direct Translation 3.**

Poniższe pytania dotyczą różnych rodzajów zmartwień, których mogłeś doświadczyć w ciągu ostatnich siedmiu dni. W poniższych stwierdzeniach nazywamy COVID-19 „wirusem”.

Martwię się, że złapię wirusa

Obawiam się, że podstawowe zasady higieny (np. mycie rąk) nie wystarczą, aby uchronić mnie przed wirusem

Martwię się, że nasz system opieki zdrowotnej nie jest w stanie uchronić mnie przed wirusem

Martwię się, że nie mogę uchronić mojej rodziny przed wirusem

Martwię się, że nasz system opieki zdrowotnej nie będzie w stanie ochronić moich bliskich

Martwię się, że dystans społeczny nie wystarczy, aby uchronić mnie przed wirusem

Martwię się, że w sklepach spożywczych skończy się żywność

Martwię się, że w sklepach spożywczych skończą się lekarstwa na przeziębieniu lub grypę

Martwię się, że w aptekach zabraknie leków na receptę

Martwię się, że w sklepach spożywczych skończy się woda

Martwię się, że w sklepach spożywczych kończą się środki czyszczące lub dezynfekujące

Martwię się, że sklepy spożywcze zostaną zamknięte

Martwię się, że cudzoziemcy roznoszą wirusa w moim kraju

Gdybym spotkał osobę z obcego kraju, martwiłbym się, że może mieć wirusa

Obawiam się kontaktu z obcokrajowcami, ponieważ mogą mieć wirusa

Martwię się, że cudzoziemcy rozprzestrzeniają wirusa, ponieważ nie są tak czyści jak my

Gdybym poszedł do restauracji specjalizującej się w zagranicznej żywności, obawiałbym się, że złapię wirusa

Gdybym był w windzie z grupą obcokrajowców, martwiłbym się, czy nie są zarażeni wirusem

Martwię się, że ludzie wokół mnie zarażą mnie wirusem

Martwię się, że jakbym dotknął czegoś w przestrzeni publicznej (np. poręczy, klamki) to złapałbym wirusa

Martwię się, że gdyby ktoś obok mnie kaszlnął lub kichnął, zaraziłbym się wirusem

Martwię się, że mogę złapać wirusa posługując się pieniędzmi lub korzystania z bankomatu

Obawiam się zmian w transakcjach gotówkowych

Martwię się, że moja poczta została skażona przez osoby obsługujące pocztę

W poniższych stwierdzeniach nazywamy COVID-19 „wirusem”. Przeczytaj każde stwierdzenie i wskaż, jak często każdy problem występował w ciągu ostatnich siedmiu dni.

Miałem problemy ze snem, ponieważ martwiłem się wirusem

Miałem złe sny o wirusie

Myślałem o wirusie, kiedy nie miałem takiego zamiaru

Wbrew mojej woli pojawiły się w moim umyśle niepokojące obrazy dotyczące wirusa

Miałem problemy z koncentracją, ponieważ ciągle myślałem o wirusie

Przypomnienia o wirusie spowodowały, że miałem reakcje fizyczne, takie jak pocenie się lub kołatanie serca

Poniższe punkty dotyczą sprawdzania zachowań. Jak często w ciągu ostatnich siedmiu dni sprawdzałeś poniższe informacje ze względu na obawy dotyczące COVID-19?

Posty w mediach społecznościowych dotyczące COVID-19

Filmy na YouTube o COVID-19

Poszukiwanie uspokojenia u znajomych lub rodziny w związku z COVID-19

Sprawdzanie własnego ciała pod kątem oznak infekcji (np. mierzenie temperatury)

Proszenie pracowników służby zdrowia (np. lekarzy lub farmaceutów) o poradę na temat COVID-19

Przeszukałem Internet w poszukiwaniu metod leczenia COVID-19

**The Polish version of the CSS Submitted to The Back-Translation**

Poniższe stwierdzenia dotyczą różnych obaw, których mógł/ła Pan/i doświadczyć w ciągu ostatnich 7 dni. W poniższych stwierdzeniach COVID-19 określany jest słowem wirus.

Obawiam się, że złapię wirusa

Obawiam się, że podstawowa higiena (np. mycie rąk) nie wystarczą, aby uchronić mnie przed wirusem

Obawiam się, że nasz system opieki zdrowotnej nie jest w stanie ochronić mnie przed wirusem

Obawiam się, że nie potrafię ochronić mojej rodziny przed wirusem

Obawiam się, że nasz system opieki zdrowotnej nie będzie w stanie ochronić moich bliskich

Obawiam się, że dystans społeczny nie wystarczy mi, aby uchronić mnie przed wirusem

Obawiam się, że w sklepach zabraknie żywności

Obawiam się, że w sklepach zabraknie leków na przeziębienie lub grypę

Obawiam się, że w aptekach zabraknie leków na receptę

Obawiam się, że w sklepach zabraknie wody

Obawiam się, że w sklepach zabraknie środków myjących lub dezynfekujących

Obawiam się, że sklepy zostaną zamknięte

Obawiam się, że obcokrajowcy rozprzestrzeniają wirusa w moim kraju

Gdybym spotkał osobę z innego kraju, obawiałbym się, że może być zarażony wirusem

Obawiam się kontaktu obcokrajowcami, ponieważ mogą być zarażeni wirusem

Obawiam się, że obcokrajowcy rozprzestrzeniają wirusa, ponieważ nie dbają o higienę tak, jak my

Gdybym poszedł do restauracji serwującej dania z innych krajów, obawiałbym się, że złapię wirusa

Gdybym był w windzie z grupą obcokrajowców, obawiałbym się, że są oni zarażeni wirusem

Obawiam się, że ludzie wokół mnie zarażą mnie wirusem

Obawiam się, że jeśli dotknąłbym czegoś w miejscu publicznym (np. poręczy, klamki), złapałbym wirusa

Obawiam się, że gdyby ktoś obok mnie zakaszlał lub kichnął, złapał(a)bym wirusa

Obawiam się, że mógłbym złapać wirusa, płacąc gotówką lub kartą

Obawiam się przyjmowania reszty w gotówce

Obawiam się, że moje przesyłki pocztowe zostały zainfekowane przez osoby obsługujące pocztę

Przeczytaj każde stwierdzenie i wskaż, jak często dany problem występował u Pana/i w ciągu ostatnich 7 dni.

Miałem problemy ze snem, ponieważ martwiłem się wirusem

Miałem złe sny o wirusie

Myślałem o wirusie, kiedy nawet tego nie chciałem

Wbrew mojej woli, w mojej głowie pojawiały się niepokojące wyobrażenia dotyczące wirusa

Miałem problemy z koncentracją, ponieważ ciągle myślałem o wirusie

Wzmianki o wirusie powodowały u mnie takie fizyczne reakcje, jak pocenie się lub przyspieszone bicie serca

Poniższe zdania dotyczą zachowań związanych ze sprawdzaniem. W ciągu ostatnich 7 dni, jak często sprawdzał Pan/i poniższe rzeczy z powodu obaw związanych z COVID

Posty w mediach społecznościowych dotyczące COVID

Filmy na YouTube dotyczące COVID

Poszukiwanie podniesienia na duchu ze strony znajomych i rodziny w związku z COVID

Sprawdzanie własnego ciała w poszukiwaniu oznak infekcji (np. mierzenie temperatury)

Proszenie pracowników służby zdrowia (np. lekarzy lub farmaceutów) o poradę w związku COVID

Poszukiwanie w Internecie informacji dotyczących leczenia COVID

**Back Translation 1.**

The following statements address various concerns you may have experienced in the last 7 days. In the following statements, COVID-19 is referred to by the word 'virus'.

I'm afraid I'm going to catch the virus

I'm afraid that basic hygiene (e.g. hand washing) is not enough to protect me from the virus

I'm afraid that our health care system is not able to protect me from the virus

I'm afraid I can't protect my family from the virus

I'm afraid that our health care system is not able to protect my loved ones from the virus

I'm afraid that social distancing is not enough to protect me from the virus

I'm afraid that shops will run out of food

I'm afraid that shops will run out of cold or flu medications

I'm afraid that pharmacies will run out of prescription drugs

I'm afraid that shops will run out of water

I'm afraid that shops will run out of cleaning agents or disinfectants

I'm afraid that shops will be closed

I'm afraid that foreigners are spreading the virus in my country

If I met a person from another country, I would be afraid that they might be infected with the virus

I'm afraid of meeting foreigners because they may be infected with the virus

I'm afraid the foreigners are spreading the virus because they don't care about hygiene like we do

If I went to a restaurant serving food from other countries, I would be afraid of catching the virus

If I were in an elevator with a group of foreigners, I would be afraid that they were infected with the virus

I'm afraid the people around me will infect me with the virus

I'm afraid that if I touched something in a public place (e.g. a handrail, door handle, doorknob), I would catch the virus

I'm afraid that if someone next to me coughed or sneezed, I would catch the virus

I'm afraid I might catch the virus when paying in cash or by card

I'm afraid to accept the change in cash

I'm afraid my mail parcels were infected by mail handlers

Read each statement and indicate how often you experienced the problem described below in the last 7 days.

I had trouble sleeping because I was concerned about the virus

I had nightmares about the virus

I thought about the virus even when I didn't want it

Against my will, disturbing images concerning the virus kept popping up in my head

I had trouble concentrating because I kept thinking about the virus

The mention of the virus caused such physical reactions as sweating or rapid heartbeat

The following sentences concern checking-related behaviours. How often did you check the following things due to concerns about COVID in the last 7 days?

Social media posts concerning COVID

YouTube videos concerning COVID

Seeking spiritual comfort in friends and family due to COVID

Checking one's own body for signs of infection (e.g. measuring temperature)

Asking health care professionals (e.g. doctors or pharmacists) for advice regarding COVID

Searching the Internet for information concerning COVID treatment

**Back Translation 2.**

The following statements relate to various concerns you may have experienced in the last 7 days. In the following statements, COVID-19 is referred to as the virus.

I am afraid I'm going to catch the virus

I am afraid that basic hygiene (e.g. washing my hands) will not be enough to protect me from the virus

I am afraid that our healthcare system is unable to protect me from the virus

I am afraid I cannot protect my family from the virus

I am afraid that our healthcare system will not be able to protect my loved ones

I am afraid that social distancing is not enough to protect me from the virus

I am afraid that the shops will run out of food

I am afraid there will be a shortage of cold or flu medicines in the shops

I am afraid that pharmacies will run out of prescription drugs

I am afraid that the shops will run out of water

I am afraid there will be a shortage of cleaning or disinfecting products in the shops

I am afraid that shops will close

I am afraid that foreigners are spreading the virus in my country

If I met a person from another country, I would be afraid that they might be infected with the virus

I fear contact with foreigners because they may be infected with the virus

I am afraid that foreigners are spreading the virus because they do not care about hygiene as much as we do

If I went to a restaurant serving food from other countries, I would be afraid of catching the virus

If I were in a lift with a group of foreigners, I would be afraid that they were infected with the virus

I am afraid that people around me will infect me with the virus

I am afraid that if I touched something in a public place (e.g. a handrail, a door handle), I would catch the virus

I am afraid that if someone coughed or sneezed near me, I would catch the virus

I am afraid I might catch the virus if I pay by cash or card

I am afraid to accept change in cash

I am afraid my mail has been infected by postal handlers

Read each statement and indicate how often the problem has occurred for you in the last 7 days.

I have had trouble sleeping because I was worried about the virus

I have had bad dreams about the virus

I have been thinking about the virus even when I didn't want to

Against my will, disturbing images of the virus have kept popping up in my head

I have had trouble concentrating because I kept thinking about the virus

Mentions of the virus have caused me to have such physical reactions as sweating or an accelerated heartbeat

The following sentences relate to checking behaviours. In the last 7 days, how often have you checked the following items because of concerns about COVID

Social media posts about COVID

YouTube videos on COVID

Seeking reassurance from friends and family in relation to COVID

Checking your own body for signs of infection (e.g. taking your temperature)

Asking healthcare professionals (e.g. doctors or pharmacists) for advice in relation to COVID

Searching online for information on COVID treatments

I am most afraid of the coronavirus

**Back Translation 3.**

The statements below refer to different fears you might have experienced over the last 7 days. In the statements below, COVID-19 is designated as “the virus”.

I am afraid of catching the virus

I am afraid basic hygiene (such as washing hands) will not be enough to protect me against the virus

I am afraid our healthcare system will not be able to protect me against the virus

I am afraid I cannot protect my family against the virus

I am afraid our healthcare system will not be able to protect my friends and family

I am afraid social distancing will not be enough to protect me against the virus

I am afraid the stores will run out of food

I am afraid the pharmacies will run out of cold and flu medications

I am afraid the pharmacies will run out of prescription medications

I am afraid the stores will run out of water

I am afraid the stores will run out of cleaning and disinfection products

I am afraid the stores will be closed

I am of foreigners spreading the virus in my country

If I met a person from another country, I would be afraid they might be infected with the virus

I am afraid of contact with foreigners because they might be infected with the virus

I am afraid the foreigners are spreading the virus because they do not care about their hygiene as we do

If I went to a restaurant serving dishes from other countries, I would be afraid of catching the virus

If I was in a lift with a group of foreigners, I would be afraid they are infected with the virus

I am afraid people around me will infect me with the virus

I am afraid that if I touch something in a public place (such as a rail or handle), I may catch the virus

I am afraid that if someone coughs or sneezes next to me, I may catch the virus

I am afraid I could catch the virus while paying with cash or card

I am afraid of receiving the change in cash

I am afraid my mail was infected by people delivering it

Read each statement and mark how often you have experienced the given issue during the last 7 days.

I had sleeping disorders because of fear of the virus

I had bad dreams about the virus

I thought about the virus even when I did not want to

Disturbing thoughts about the virus were appearing in my head against my will

I had trouble focusing because I could not stop thinking about the virus

I experienced physical reactions, such as sweating or faster heart rate, whenever the virus was mentioned

The statements below refer to monitoring. How often have you been checking the following things during the last 7 days due to your fear of COVID?

Social media posts about COVID

YouTube videos about COVID

Turning to my friends and family to lift up my spirits because of COVID

Checking my body temperature looking for signs of infection (e.g., measuring the temperature)

Asking healthcare professionals (e.g., doctors or pharmacists) for advice about COVID

Searching the Internet for information on COVID treatment.

**The Polish Experimental Version of the CSS Employed in a Study With Bilingual Participants**

**Poniższa ankieta dotyczy różnych obaw, których mogła Pani/mógł Pan doświadczyć w ciągu ostatnich siedmiu dni. W poniższych stwierdzeniach, używamy słowa „wirus” w odniesieniu do COVID-19.**

Wcale

Trochę

Umiarkowanie

Bardzo

Niezmiernie

Boję się, że zarażę się wirusem

Obawiam się, że podstawowa higiena (np. mycie rąk) nie wystarczy, aby uchronić mnie przed zarażeniem się wirusem

Obawiam się, że nasz system ochrony zdrowia nie jest w stanie uchronić mnie przed wirusem

Obawiam się, że nie jestem w stanie uchronić mojej rodziny przed wirusem

Obawiam się, że nasz system ochrony zdrowia nie jest w stanie chronić moich najbliższych przed wirusem

Obawiam się, że utrzymywanie społecznego dystansu to za mało, aby uchronić mnie przed wirusem

Obawiam się, że wystąpi problem z dostępnością jedzenia w sklepach spożywczych

Obawiam się, że zabraknie lekarstw na przeziębienie i grypę w sklepach spożywczych

Obawiam się, że w aptekach będzie problem z dostępnością lekarstw na receptę

Obawiam się, że w sklepach spożywczych będzie kłopot z dostępnością wody pitnej

Obawiam się, że w sklepach i marketach będzie problem z dostępnością środków czystości lub dezynfekujących

Obawiam się, że sklepy zostaną zamknięte

Obawiam się, że obcokrajowcy roznoszą wirusa w moim kraju

Jeśli spotkałabym/spotkałbym obcokrajowca, miałabym/miałbym obawy, że może mieć wirusa

Obawiam się, że będę miał/a kontakt z obcokrajowcem, ponieważ oni mogą mieć wirusa

Obawiam się, że obcokrajowcy roznoszą wirusa, ponieważ ich standardy higieny osobistej nie są na takim poziomie jak nasze

Jeśli poszłabym/poszedłbym do restauracji z kuchnią zagraniczną, obawiałbym/obawiałabym się, że zarażę się wirusem

Jeśli znalazłabym/znalazłbym się w windzie z grupą obcokrajowców, obawiałbym się/obawiałabym się, że mogą być zakażeni wirusem

Obawiam się, że ludzie, z którymi przebywam, zarażą mnie wirusem

Obawiam się, że dotykając przedmiotów ogólnodostępnych (np. poręcz, klamka), zarażę się wirusem

Obawiam się, że zarażę się wirusem od kogoś, kto kichnie lub zakaszle w pobliżu mnie

Obawiam się, że mogę zarazić się wirusem, posługując się gotówką lub korzystając z bankomatu

Obawiam się przyjmować resztę w gotówce

Obawiam się, że moja poczta mogła zostać skażona przez osoby obsługujące pocztę

**W poniższych stwierdzeniach używamy słowa „wirus” w odniesieniu do COVID-19. Proszę przeczytać każde stwierdzenie i wskazać, jak często pojawiał się u Pani/Pana dany problem w przeciągu ostatnich siedmiu dni.**

Miałem/miałam problemy z zasypianiem z powodu obaw przed wirusem

Miałem/miałam koszmary związane z wirusem

Myślałem/myślałam o wirusie, kiedy nie miałem/miałam takiego zamiaru

Miałem/miałam niepokojące wyobrażenia na temat wirusa wbrew mojej woli

Miałem/miałam problemy z koncentracją, ponieważ myślałem/myślałam o wirusie

Wzmianki o wirusie powodują u mnie takie reakcje, jak pocenie się lub przyśpieszone bicie serca

Nigdy

Rzadko

Czasami

Często

Prawie zawsze

**Poniższe pytania odnoszą się do zachowań związanych ze sprawdzaniem i szukaniem informacji. W związku z COVID-19, jak często w przeciągu ostatnich siedmiu dni:**

Nigdy

Rzadko

Czasami

Często

Prawie zawsze

Sprawdzałeś/sprawdzałaś posty odnośnie do COVID-19 w mediach społecznościowych?

Oglądałeś/oglądałaś widea na YouTube o tematyce COVID-19?

Szukałeś/szukałaś wsparcia od znajomych i rodziny w związku z COVID-19?

Sprawdzałeś/sprawdzałaś swój organizm pod kątem infekcji (np. mierzenie temperatury)?

Prosiłeś/prosiłaś lekarzy, farmaceutów lub innych specjalistów z zakresu ochrony zdrowia o radę na temat COVID-19?

Przeszukiwałeś/przeszukiwałaś Internet pod kątem leczenia COVID-19?

**The Polish Pre-final Version of the CSS Employed in a Pilot Study**

**Poniższa ankieta dotyczy różnych obaw, których mogła Pani/mógł Pan doświadczyć
w ciągu ostatnich siedmiu dni. W poniższych stwierdzeniach, używamy słowa „wirus”
w odniesieniu do COVID-19.**

Wcale

Trochę

Umiarkowanie

Bardzo

Niezmiernie

1. Boję się, że zakażę się wirusem.
2. Obawiam się, że podstawowa higiena (np. mycie rąk) nie wystarczy, aby uchronić mnie przed wirusem.
3. Obawiam się, że nasz system ochrony zdrowia nie jest w stanie uchronić mnie przed wirusem.
4. Obawiam się, że nie jestem w stanie ochronić mojej rodziny przed wirusem
5. Obawiam się, że nasz system ochrony zdrowia nie jest w stanie chronić moich najbliższych przed wirusem.
6. Obawiam się, że utrzymywanie społecznego dystansu to za mało, aby uchronić mnie przed wirusem.
7. Obawiam się, że mogą wystąpić problemy z dostawami żywności do sklepów spożywczych.
8. Obawiam się, że w sklepach spożywczych zabraknie lekarstw na przeziębienie i grypę
9. Obawiam się, że w aptekach mogą wystąpić problemy z dostępnością lekarstw na receptę.
10. Obawiam się, że w sklepach spożywczych może zabraknąć wody pitnej.
11. Obawiam się, że w sklepach i marketach będzie problem z dostępnością środków czystości lub dezynfekujących.
12. Obawiam się, że sklepy spożywcze zostaną zamknięte.
13. Obawiam się, że obcokrajowcy roznoszą wirusa w Polsce.
14. Jeśli spotkałabym/spotkałbym obcokrajowca, miałabym/miałbym obawy, że osoba ta może być zakażona wirusem.
15. Boję się nawiązywać kontaktów z obcokrajowcami, ponieważ mogą oni być zakażeni wirusem.
16. Obawiam się, że obcokrajowcy roznoszą wirusa, ponieważ ich standardy higieny osobistej są na niższym poziomie niż nasze.
17. Obawiałabym/obawiałbym się iść do restauracji z kuchnią zagraniczną, bo można tam zakazić się wirusem.
18. Jeśli znalazłabym/znalazłbym się w windzie z grupą obcokrajowców, obawiałbym się/obawiałabym się, że mogą być zakażeni wirusem.
19. Obawiam się, że ludzie wokół mnie zarażą mnie wirusem.
20. Obawiam się, że dotykając przedmiotów znajdujących się w miejscach. ogólnodostępnych (np. poręcze, klamki), mogę zarazić się wirusem.
21. Obawiam się, że zarażę się wirusem od kogoś, kto kichnie lub zakaszle w pobliżu mnie.
22. Obawiam się, że mogę zarazić się wirusem, posługując się gotówką lub korzystając
    z bankomatu.
23. Obawiam się przyjmować resztę w gotówce.
24. Obawiam się, że moja poczta mogła zostać skażona przez osoby obsługujące pocztę.

**W poniższych stwierdzeniach używamy słowa „wirus” w odniesieniu do COVID-19. Proszę przeczytać każde stwierdzenie i wskazać, jak często pojawiał się u Pani/Pana dany problem w przeciągu ostatnich siedmiu dni.**

Nigdy

Rzadko

Czasami

Często

Prawie zawsze

1. Z powodu obaw przed wirusem miałem/miałam problemy ze snem.
2. Miałem/miałam koszmary nocne związane z wirusem.
3. Myślałem/myślałam o wirusie, nawet jeśli nie miałem/miałam takiego zamiaru.
4. Niezależnie od mojej woli atakowały mnie niepokojące wyobrażenia na temat wirusa.
5. Miałem/miałam problemy z koncentracją, ponieważ stale myślałem/myślałam
   o wirusie.
6. Zdarzało się, że wzmianki o wirusie powodowały u mnie takie reakcje, jak pocenie się lub przyśpieszone bicie serca.

**Poniższe pytania odnoszą się do zachowań związanych ze sprawdzaniem i szukaniem informacji. W związku z COVID-19, jak często w przeciągu ostatnich siedmiu dni:**

Nigdy

Rzadko

Czasami

Często

Prawie zawsze

1. Sprawdzałeś/sprawdzałaś posty odnośnie do COVID-19 w mediach społecznościowych?
2. Oglądałeś/oglądałaś filmiki na YouTube o tematyce związanej z COVID-19?
3. Szukałeś/szukałaś u znajomych i rodziny wsparcia lub pocieszenia w związku z COVID-19?
4. Badałeś/badałaś swój organizm pod kątem infekcji (np. mierzenie temperatury)?
5. Prosiłeś/prosiłaś lekarzy, farmaceutów lub innych pracowników ochrony zdrowia o poradę na temat COVID-19?

**The Final Polish Version of the CSS Employed in the Validation Study**

**Poniższa ankieta dotyczy różnych obaw, których mogła Pani/mógł Pan doświadczyć w ciągu ostatnich siedmiu dni. W poniższych stwierdzeniach,  używamy słowa „wirus” w odniesieniu do COVID-19.**

0 Wcale

1 Trochę

2 Umiarkowanie

3 Bardzo

4 Niezmiernie

1. Boję się, że zakażę się wirusem
2. Obawiam się, że podstawowa higiena (np.  mycie rąk) nie wystarczy, aby uchronić mnie przed wirusem
3. Obawiam się, że nasz system ochrony zdrowia nie jest w stanie uchronić mnie przed wirusem
4. Obawiam się, że nie jestem w stanie ochronić mojej rodziny przed wirusem
5. Obawiam się, że nasz system ochrony zdrowia nie jest w stanie chronić moich najbliższych przed wirusem
6. Obawiam się, że utrzymywanie społecznego dystansu to za mało, aby uchronić mnie przed wirusem
7. Obawiam się, że mogą wystąpić problemy z dostawami żywności do sklepów spożywczych
8. Obawiam się, że w sklepach spożywczych zabraknie lekarstw na przeziębienie i grypę
9. Obawiam się, że w aptekach mogą wystąpić problemy z dostępnością lekarstw na receptę
10. Obawiam się, że w sklepach spożywczych może zabraknąć wody pitnej.
11. Obawiam się, że w sklepach i marketach będzie problem z dostępnością środków czystości lub dezynfekujących
12. Obawiam się, że sklepy spożywcze zostaną zamknięte
13. Obawiam się, że obcokrajowcy roznoszą wirusa w Polsce
14. Jeśli spotkałabym/spotkałbym obcokrajowca, miałabym/miałbym obawy, że osoba ta może być zakażona wirusem
15. Boję się nawiązywać kontaktów z obcokrajowcami, ponieważ mogą oni być zakażeni wirusem
16. Obawiam się, że obcokrajowcy roznoszą wirusa, ponieważ ich standardy higieny osobistej są na niższym poziomie niż nasze
17. Obawiałabym/obawiałbym się iść do restauracji z kuchnią zagraniczną, bo można tam zakazić się wirusem
18. Jeśli znalazłabym/znalazłbym się w windzie z grupą obcokrajowców, obawiałbym się/obawiałabym się, że mogą być zakażeni wirusem
19. Obawiam się, że ludzie wokół mnie zarażą mnie wirusem
20. Obawiam się, że dotykając przedmiotów znajdujących się w miejscach ogólnodostępnych (np. poręcze, klamki), mogę zarazić się wirusem
21. Obawiam się, że zarażę się wirusem od kogoś, kto kichnie lub zakaszle w pobliżu mnie
22. Obawiam się, że mogę zarazić się wirusem, posługując się gotówką lub korzystając z bankomatu
23. Obawiam się przyjmować resztę w gotówce
24. Obawiam się, że moja poczta mogła zostać skażona przez osoby obsługujące pocztę

**W poniższych stwierdzeniach używamy słowa „wirus” w odniesieniu do COVID-19. Proszę przeczytać każde stwierdzenie i wskazać, jak często pojawiał się u Pani/Pana dany problem w przeciągu ostatnich siedmiu dni.**

Nigdy

Rzadko

Czasami

Często

Prawie zawsze

1. Z powodu obaw przed wirusem miałem/miałam problemy ze snem
2. Miałem/miałam koszmary nocne związane z wirusem
3. Myślałem/myślałam o wirusie, nawet jeśli nie miałem/miałam takiego zamiaru
4. Niezależnie od mojej woli atakowały mnie niepokojące wyobrażenia na temat wirusa
5. Miałem/miałam problemy z koncentracją, ponieważ stale myślałem/myślałam o wirusie
6. Zdarzało się, że wzmianki o wirusie powodowały u mnie takie reakcje, jak pocenie się lub przyśpieszone bicie serca

**Poniższe pytania odnoszą się do zachowań związanych ze sprawdzaniem i szukaniem informacji. W związku z COVID-19, jak często w przeciągu ostatnich siedmiu dni:**

Nigdy

Rzadko

Czasami

Często

Prawie zawsze

1. Sprawdzałeś/sprawdzałaś posty odnośnie do COVID-19 w mediach społecznościowych?
2. Oglądałeś/oglądałaś filmiki na YouTube o tematyce związanej z COVID-19?
3. Szukałeś/szukałaś u znajomych i rodziny wsparcia lub pocieszenia w związku z COVID-19?
4. Badałeś/badałaś swój organizm pod kątem infekcji (np. mierzenie temperatury)?
5. Prosiłeś/prosiłaś lekarzy, farmaceutów lub innych pracowników ochrony zdrowia o poradę na temat COVID-19?
6. Wyszukiwałeś/wyszukiwałaś w Internecie informacji na temat leczenia COVID-19?
