# Supplementary material for: The Polish COVID Stress Scales: Considerations of psychometric functioning, measurement invariance, and validity
Source: PLoS One. 2021 Dec 1;16(12):e0260459. doi: 10.1371/journal.pone.0260459 (PMC8635383; doi:10.1371/journal.pone.0260459)
Supplement: S1 Table — The COVID-19 Stress Scales (CSS) (Taylor et al., 2020a) were translated following the recommendations of the “ISPOR Task Force for Translation and Cultural Adaptation” (Wild et al., 2005) using a 10-step procedure for translation. (DOCX) [file pone.0260459.s003.docx]

| **S1 Table**  *Steps in the Translation Procedure of the Original COVID-19 Stress Scales (CSS) into the Polish-language* |
| --- |
| Step |
| Permission from the authors of the original CSS to use the instrument and elaborate its Polish translation and validation was obtained. Three independent professional translators, native Polish speakers who were fluent in English were determined.  The forward translations of the original CSS were developed by three independent professional translators, native Polish speakers.  Reconciliation of three forward translations into a single forward translation.  The backward translation process was performed and the Polish version of the CSS was translated back into English by another three independent professional translators.  Review of the back translation by comparing the back-translated version of the Polish CSS and the original CSS to ensure the conceptual  equivalence of the translation and identify discrepancies between the original CSS and the reconciled Polish translation.  To detect and deal with any discrepancies between the back-translated version of the CSS and the original CSS, the items of the Polish version of the CSS were harmonized.  Cognitive debriefing of the experimental version of the Polish CSS was performed by assessing the Polish CSS in a small sample of 38 bilingual university students in order to evaluate the comprehensibility and cognitive equivalence of the Polish CSS, as well as to detect any inappropriate or confusing items and issues.  The review of the results obtained through cognitive debriefing and incorporation of these findings to improve and finalize the translation.  The final proofreading of the Polish CSS’ items was performed to detect and correct any errors (typographic, grammatical, or other).  A final report documenting the translation process of the Polish CSS was prepared at the end of the process. |
| *Note*. The *COVID-19 Stress Scales (CSS)* (Taylor et al., 2020a) were translated following the recommendations of the “ISPOR Task Force for Translation and Cultural Adaptation” (Wild et al., 2005) using a 10-step procedure for translation. |
