# Supplementary material for: The Polish COVID Stress Scales: Considerations of psychometric functioning, measurement invariance, and validity
Source: PLoS One. 2021 Dec 1;16(12):e0260459. doi: 10.1371/journal.pone.0260459 (PMC8635383; doi:10.1371/journal.pone.0260459)
Supplement: S2 Table — M = mean; SD = standard deviation; t = t-test; p = p-value; r = Pearson’s correlation between the Polish and the corresponding English items. * p < .05. ** p < .01. (DOCX) [file pone.0260459.s004.docx]

| **S2 Table**  *Descriptive and Cross-language Comparison of the Original CSS Item Scores and the CSS-PL_EX in the Study 1 with Bilingual Participants* | | | | | | | | |
| --- | --- | --- | --- | --- | --- | --- | --- | --- |
| Items | English version of the CSS | | | Polish version of the CSS | |  |  |  |
|  | *M* | *SD* | | *M* | *SD* | *t*(37) | *p* | *r* |
| CSS1 | 1.76 | 0.85 | | 1.61 | 0.92 | 1.64 | .110 | .78*** |
| CSS2 | 2.05 | 0.99 | | 1.76 | 1.00 | 2.06 | .047 | .62*** |
| CSS3 | 2.84 | 1.05 | | 2.58 | 1.11 | 1.53 | .133 | .52** |
| CSS4 | 2.71 | 1.1 | | 2.26 | 1.31 | 2.99 | .005 | .66*** |
| CSS5 | 3.13 | 0.99 | | 2.84 | 0.89 | 1.87 | .070 | .49** |
| CSS6 | 1.74 | 0.95 | | 1.82 | 1.09 | - 0.45 | .653 | .45** |
| CSS7 | 0.87 | 1.14 | | 0.45 | 0.8 | 2.16 | .037 | .28 |
| CSS8 | 0.79 | 0.96 | | 0.45 | 0.72 | 3.15 | .003 | .72*** |
| CSS9 | 1.18 | 1.27 | | 0.79 | 0.96 | 2.5 | .017 | .65*** |
| CSS10 | 0.68 | 1.17 | | 0.37 | 0.68 | 1.78 | .083 | .39* |
| CSS11 | 1.00 | 1.19 |  | 0.95 | 1.06 | 0.32 | .750 | .60*** |
| CSS12 | 1.32 | 1.3 |  | 1.32 | 1.14 | 0.00 | 1.000 | .35* |
| CSS13 | 0.71 | 0.93 |  | 0.37 | 0.59 | 2.7 | .010 | .55*** |
| CSS14 | 0.74 | 0.83 |  | 0.63 | 0.79 | 0.85 | .401 | .55*** |
| CSS15 | 0.68 | 0.81 |  | 0.26 | 0.6 | 4.04 | .000 | .62*** |
| CSS16 | 0.03 | 0.16 |  | 0.00 | 0.00 | 1.0 | .324 | . |
| CSS17 | 0.26 | 0.6 |  | 0.39 | 0.92 | -1.09 | .281 | .59*** |
| CSS18 | 0.89 | 0.95 |  | 0.84 | 1.15 | 0.35 | .729 | .63*** |
| CSS19 | 1.68 | 0.93 |  | 1.29 | 0.98 | 2.57 | .014 | .51* |
| CSS20 | 1.87 | 1.17 |  | 1.79 | 0.99 | 0.41 | .686 | .40* |
| CSS21 | 2.37 | 1.15 |  | 2.37 | 1.1 | 0.00 | 1.000 | .70*** |
| CSS22 | 1.58 | 1.11 |  | 1.39 | 1.2 | 1.0 | .324 | .52* |
| CSS23 | 1.32 | 1.09 |  | 1.08 | 1.17 | 1.71 | .095 | .72*** |
| CSS24 | 0.76 | 0.91 |  | 0.68 | 0.93 | 0.52 | .608 | .48** |
| CSS25 | 0.82 | 0.98 |  | 0.37 | 0.85 | 3.09 | .004 | .54** |
| CSS26 | 0.53 | 0.95 |  | 0.21 | 0.47 | 2.23 | .032 | .41* |
| CSS27 | 1.45 | 1.06 |  | 1.16 | 1.08 | 1.87 | .070 | .60*** |
| CSS28 | 0.92 | 1.1 |  | 1.13 | 1.23 | -1.6 | .118 | .77*** |
| CSS29 | 1.11 | 1.18 |  | 0.68 | 0.96 | 2.35 | .024 | .48** |
| CSS30 | 0.95 | 1.41 |  | 0.5 | 0.86 | 2.07 | .045 | .40* |
| CSS31 | 1.45 | 1.22 |  | 1.45 | 1.08 | 0.00 | 1.000 | .60*** |
| CSS32 | 0.42 | 0.86 |  | 0.5 | 0.73 | -0.77 | .446 | .69*** |
| CSS33 | 1.13 | 1.12 |  | 0.79 | 0.88 | 2.12 | .041 | .53** |
| CSS34 | 1.05 | 1.37 |  | 1.08 | 1.08 | -0.13 | .898 | .49** |
| CSS35 | 0.34 | 0.75 |  | 0.24 | 0.68 | 0.94 | .353 | .53** |
| CSS36 | 0.53 | 0.95 |  | 0.82 | 1.04 | -1.68 | .102 | .43** |
| *Note*. *M* = mean; *SD* = standard deviation; *t* = *t*-test; *p* = *p*-value; *r* = Pearson’s correlation between the Polish and the corresponding English items. ** p* < .05. ** *p* < .01. | | | | | | | | |
