# Supplementary material for: The Polish COVID Stress Scales: Considerations of psychometric functioning, measurement invariance, and validity
Source: PLoS One. 2021 Dec 1;16(12):e0260459. doi: 10.1371/journal.pone.0260459 (PMC8635383; doi:10.1371/journal.pone.0260459)
Supplement: S3 Table — M = mean; SD = standard deviation; avg. rit = average item-total correlation; t = t-test; r = Pearson’s correlation between the subscales’ scores of the CSS-PL_EX and the original CSS. ** p < .001, two—tailed. * p < .05 two—tailed. (DOCX) [file pone.0260459.s005.docx]

| **S3 Table**  *Descriptive, Internal Consistency Reliability, and Cross-Language Comparison of the Polish Experimental Version of the CSS (CSS-PL_EX) and the Original CSS Scales Scores in Study 1* | | | | | | | | | | |
| --- | --- | --- | --- | --- | --- | --- | --- | --- | --- | --- |
|  | Polish experimental version of the CSS | | | | English version of the CSS | | | |  |  |
| Subscales CSS | *M* | *SD* | McDonald *ω* | avg._it_ | *M* | *SD* | McDonald *ω* | avg._it_ | *t*(37) | *r* |
| COVID danger and contamination | 1.79 | 0.77 | .92 | .74 | 1.98 | 0.69 | .88 | .67 | 2.00 | .67*** |
| COVID socioeconomic consequences | 0.72 | 0.73 | .90 | .82 | 0.97 | 0.99 | .92 | .85 | 1.93 | .59** |
| COVID xenophobia | 0.42 | 0.48 | .71 | .56 | 0.55 | 0.56 | .87 | .73 | 2.01 | .69** |
| COVID traumatic stress symptoms | 0.68 | 0.76 | .89 | .82 | 0.96 | 0.89 | .88 | .79 | 2.62* | .68** |
| COVID compulsive checking | 0.81 | 0.64 | .79 | .68 | 0.82 | 0.81 | .85 | .76 | 0.09 | .71** |
| *Note*. *M* = mean; *SD* = standard deviation; avg. *r*_it_ = average item-total correlation; *t* = *t*-test; *r* = Pearson’s correlation between the subscales’ scores of the CSS-PL_EX and the original CSS. ** *p* < .001, two—tailed. * *p* < .05 two—tailed. | | | | | | | | | | |
