# Supplementary material for: The Polish COVID Stress Scales: Considerations of psychometric functioning, measurement invariance, and validity
Source: PLoS One. 2021 Dec 1;16(12):e0260459. doi: 10.1371/journal.pone.0260459 (PMC8635383; doi:10.1371/journal.pone.0260459)
Supplement: S4 Table — CSS = COVID Stress Scale Item; F1 = Factor 1. Models fit using weighted least squares with mean and variance adjustment (WLSMV) estimation. Factor loadings greater than λ = ±.40 presented in bold. (DOCX) [file pone.0260459.s006.docx]

| **S4 Table** | | | |
| --- | --- | --- | --- |
| *Factor Loadings From 1 Factor Exploratory Structural Equation Model* | | | |
|  | Polish sample | Dutch sample |  |
|  | F1 | F1 |  |
| CSS-1 | **.80** | **.68** |  |
| CSS-2 | **.75** | **.73** |  |
| CSS-3 | **.78** | **.68** |  |
| CSS-4 | **.73** | **.73** |  |
| CSS-5 | **.76** | **.77** |  |
| CSS-6 | **.70** | **.67** |  |
| CSS-7 | **.54** | **.69** |  |
| CSS-8 | **.56** | **.69** |  |
| CSS-9 | **.55** | **.64** |  |
| CSS-10 | **.62** | **.80** |  |
| CSS-11 | **.62** | **.63** |  |
| CSS-12 | **.48** | **.63** |  |
| CSS-13 | **.76** | **.65** |  |
| CSS-14 | **.89** | **.85** |  |
| CSS-15 | **.91** | **.85** |  |
| CSS-16 | **.63** | **.58** |  |
| CSS-17 | **.67** | **.69** |  |
| CSS-18 | **.77** | **.72** |  |
| CSS-19 | **.87** | **.73** |  |
| CSS-20 | **.83** | **.72** |  |
| CSS-21 | **.83** | **.66** |  |
| CSS-22 | **.88** | **.85** |  |
| CSS-23 | **.85** | **.83** |  |
| CSS-24 | **.76** | **.68** |  |
| CSS-25 | **.79** | **.74** |  |
| CSS-26 | **.74** | **.78** |  |
| CSS-27 | **.80** | **.57** |  |
| CSS-28 | **.86** | **.75** |  |
| CSS-29 | **.83** | **.75** |  |
| CSS-30 | **.76** | **.82** |  |
| CSS-31 | **.50** | .37 |  |
| CSS-32 | .24 | .40 |  |
| CSS-33 | **.63** | **.53** |  |
| CSS-34 | **.55** | **.51** |  |
| CSS-35 | **.57** | **.69** |  |
| CSS-36 | **.61** | **.57** |  |
| CSS = COVID Stress Scale Item; F1 = Factor 1. Models fit using weighted least squares with mean and variance adjustment (WLSMV) estimation. Factor loadings greater than λ = ±.40 presented in **bold.** | | | |
