# Supplementary material for: The Polish COVID Stress Scales: Considerations of psychometric functioning, measurement invariance, and validity
Source: PLoS One. 2021 Dec 1;16(12):e0260459. doi: 10.1371/journal.pone.0260459 (PMC8635383; doi:10.1371/journal.pone.0260459)
Supplement: S5 Table — CSS = COVID Stress Scale Item; F1…F4 = Factors 1 through 4. Models fit using weighted least squares with mean and variance adjustment (WLSMV) estimation and targeted oblique rotation. The rotation targets for items not associated with a factor were set to 0. Factor loadings greater than λ = ±.40 presented in bold. Factor correlations ranged from r = .20 to .64 (average r = .34) in the Polish sample, and from r = .26 to .56 (average r = .37) in the Dutch sample. (DOCX) [file pone.0260459.s007.docx]

| **S5 Table** | | | | | | | | |
| --- | --- | --- | --- | --- | --- | --- | --- | --- |
| *Factor Loadings From 4 Factor Exploratory Structural Equation Model* | | | | | | | | |
|  | Polish sample | | | | Dutch sample | | | |
|  | F1 | F2 | F3 | F4 | F1 | F2 | F3 | F4 |
| CSS-1 | **.66** | -.15 | .10 | .27 | **.63** | -.12 | .06 | .20 |
| CSS-2 | **.74** | -.06 | -.01 | .14 | **.64** | -.08 | .05 | .23 |
| CSS-3 | **.91** | .26 | **-.53** | -.03 | **.67** | -.16 | -.02 | .21 |
| CSS-4 | **.76** | -.03 | -.15 | .18 | **.75** | -.26 | -.15 | .29 |
| CSS-5 | **.93** | .27 | **-.64** | -.03 | **.77** | -.26 | -.13 | .28 |
| CSS-6 | **.65** | .03 | .03 | .12 | **.64** | .00 | .01 | .15 |
| CSS-7 | -.03 | **.87** | .06 | .02 | .07 | **.71** | .04 | .23 |
| CSS-8 | -.07 | **.81** | .14 | .07 | .13 | **.74** | .00 | .18 |
| CSS-9 | .06 | **.77** | .03 | .10 | .17 | **.68** | -.07 | .18 |
| CSS-10 | -.05 | **.86** | .14 | .06 | .14 | **.70** | .08 | .31 |
| CSS-11 | .26 | **.74** | .10 | -.04 | .21 | **.55** | .05 | .18 |
| CSS-12 | -.03 | **.80** | .10 | .01 | -.05 | **.63** | .22 | .22 |
| CSS-13 | .04 | .14 | **.75** | .14 | .04 | -.12 | **.79** | .09 |
| CSS-14 | .15 | .13 | **.78** | .13 | .17 | -.14 | **.9** | -.03 |
| CSS-15 | .13 | .13 | **.80** | .14 | .08 | -.17 | **.98** | -.02 |
| CSS-16 | -.04 | .29 | **.68** | .11 | -.16 | .18 | **.80** | .03 |
| CSS-17 | .13 | .20 | **.67** | .04 | .05 | .31 | **.57** | .13 |
| CSS-18 | .26 | .01 | **.67** | .13 | .12 | -.02 | **.78** | .04 |
| CSS-19 | **.73** | -.14 | .20 | .17 | **.71** | -.13 | .24 | .01 |
| CSS-20 | **.79** | -.14 | .24 | .01 | **.76** | .08 | .11 | -.08 |
| CSS-21 | **.83** | -.19 | .14 | .07 | **.78** | -.11 | .16 | -.11 |
| CSS-22 | **.88** | .00 | .32 | -.19 | **.91** | **.44** | .00 | -.36 |
| CSS-23 | **.84** | .02 | .37 | -.22 | **.90** | **.42** | -.02 | -.36 |
| CSS-24 | **.71** | .15 | .31 | -.14 | **.58** | .35 | .12 | -.09 |
| CSS-25 | .11 | .01 | .00 | **.79** | -.06 | .04 | .14 | **.83** |
| CSS-26 | -.11 | .04 | .18 | **.83** | -.14 | .19 | .21 | **.79** |
| CSS-27 | .06 | -.03 | -.06 | **.88** | .26 | -.02 | -.21 | **.62** |
| CSS-28 | .06 | -.08 | .00 | **.91** | .19 | .00 | .01 | **.72** |
| CSS-29 | -.02 | .01 | .03 | **.91** | .13 | .01 | .00 | **.79** |
| CSS-30 | .00 | .10 | .07 | **.78** | .22 | .09 | .13 | **.64** |
| CSS-31 | .10 | .04 | -.11 | **.57** | .02 | -.06 | -.02 | **.51** |
| CSS-32 | -.31 | .16 | -.03 | **.56** | -.09 | .18 | .00 | **.51** |
| CSS-33 | .11 | -.05 | .01 | **.67** | .19 | .08 | -.03 | **.48** |
| CSS-34 | .15 | -.04 | .06 | **.50** | .05 | .08 | -.02 | **.59** |
| CSS-35 | .04 | .04 | .09 | **.59** | -.04 | **.42** | .12 | **.54** |
| CSS-36 | .06 | .05 | .04 | **.64** | -.11 | .28 | .12 | **.57** |
| CSS = COVID Stress Scale Item; F1…F4 = Factors 1 through 4. Models fit using weighted least squares with mean and variance adjustment (WLSMV) estimation and targeted oblique rotation. The rotation targets for items not associated with a factor were set to 0. Factor loadings greater than λ = ±.40 presented in **bold.** Factor correlations ranged from *r* = .20 to .64 (average *r* = .34) in the Polish sample, and from *r* = .26 to .56 (average *r* = .37) in the Dutch sample. | | | | | | | | |
