# Supplementary material for: The Polish COVID Stress Scales: Considerations of psychometric functioning, measurement invariance, and validity
Source: PLoS One. 2021 Dec 1;16(12):e0260459. doi: 10.1371/journal.pone.0260459 (PMC8635383; doi:10.1371/journal.pone.0260459)
Supplement: S6 Table — CSS = COVID Stress Scale Item; F1…F5 = Factors 1 through 5. Models fit using weighted least squares with mean and variance adjustment (WLSMV) estimation and targeted oblique rotation. The rotation targets for items not associated with a factor were set to 0. Factor loadings greater than λ = ±.40 presented in bold. Factor correlations ranged from r = .05 to .55 (average r = .28) in the Polish sample, and from r = .04 to .49 (average r = .31) in the Dutch sample. (DOCX) [file pone.0260459.s008.docx]

| **S6 Table** | | | | | | | | | | |
| --- | --- | --- | --- | --- | --- | --- | --- | --- | --- | --- |
| *Factor Loadings From 5 Factor Exploratory Structural Equation Model* | | | | | | | | | | |
|  | Polish sample | | | | | Dutch sample | | | | |
|  | F1 | F2 | F3 | F4 | F5 | F1 | F2 | F3 | F4 | F5 |
| CSS-1 | **.72** | -.13 | .13 | .16 | .05 | **.64** | -.03 | .06 | .19 | -.12 |
| CSS-2 | **.78** | -.06 | .06 | .02 | .11 | **.62** | .10 | .06 | .18 | -.18 |
| CSS-3 | **.79** | .13 | -.13 | -.23 | **.53** | **.62** | .11 | .00 | .16 | -.29 |
| CSS-4 | **.79** | -.04 | -.03 | .04 | .22 | **.66** | .07 | -.12 | .28 | -.36 |
| CSS-5 | **.81** | .14 | -.22 | -.23 | **.59** | **.66** | .15 | -.09 | .24 | **-.43** |
| CSS-6 | **.69** | .04 | .08 | .01 | .09 | **.62** | .18 | -.01 | .07 | -.18 |
| CSS-7 | -.02 | **.92** | -.05 | .02 | -.04 | .00 | **.85** | .02 | -.01 | .06 |
| CSS-8 | -.10 | **.82** | .14 | .00 | .07 | .02 | **.93** | .00 | -.05 | -.11 |
| CSS-9 | .06 | **.79** | .05 | .01 | .14 | .08 | **.89** | -.07 | -.07 | -.13 |
| CSS-10 | -.04 | **.91** | .01 | .06 | -.09 | .07 | **.83** | .06 | .06 | .10 |
| CSS-11 | .27 | **.78** | .01 | -.07 | -.04 | .17 | **.69** | .02 | -.03 | .01 |
| CSS-12 | -.03 | **.83** | .02 | .00 | -.03 | -.06 | **.70** | .19 | -.03 | .19 |
| CSS-13 | -.07 | -.01 | **.97** | -.07 | .13 | -.01 | -.01 | **.81** | .10 | -.17 |
| CSS-14 | .09 | -.01 | **.96** | -.10 | .12 | .13 | -.06 | **.91** | -.01 | -.16 |
| CSS-15 | .06 | .00 | **.97** | -.06 | .07 | .06 | -.13 | **.99** | .01 | -.1 |
| CSS-16 | -.16 | .19 | **.83** | .00 | -.03 | -.14 | .12 | **.79** | -.05 | .19 |
| CSS-17 | .04 | .11 | **.77** | -.04 | -.10 | .08 | .26 | **.54** | .00 | .24 |
| CSS-18 | .20 | -.10 | **.82** | -.02 | .00 | .11 | .02 | **.79** | .01 | -.04 |
| CSS-19 | **.78** | -.11 | .19 | .10 | -.05 | **.73** | -.10 | .24 | .03 | -.11 |
| CSS-20 | **.83** | -.06 | .08 | .07 | -.24 | **.82** | -.11 | .09 | -.05 | .15 |
| CSS-21 | **.89** | -.13 | .04 | .05 | -.12 | **.82** | -.17 | .14 | -.07 | -.05 |
| CSS-22 | **.83** | .10 | .00 | .08 | **-.44** | **.94** | .03 | -.04 | -.30 | **.41** |
| CSS-23 | **.74** | .11 | .07 | .06 | **-.48** | **.93** | -.01 | -.06 | -.29 | **.43** |
| CSS-24 | **.67** | .23 | .10 | .02 | -.35 | **.63** | .17 | .06 | -.16 | .33 |
| CSS-25 | .23 | .08 | .03 | **.66** | .12 | -.01 | .06 | .19 | **.74** | .23 |
| CSS-26 | -.03 | .08 | .22 | **.72** | .05 | -.10 | .23 | .24 | **.65** | .27 |
| CSS-27 | .20 | .05 | -.02 | **.73** | .18 | .32 | -.07 | -.17 | **.62** | .16 |
| CSS-28 | .20 | .00 | .04 | **.77** | .14 | .23 | .04 | .06 | **.66** | .12 |
| CSS-29 | .13 | .10 | .01 | **.79** | .07 | .17 | .03 | .05 | **.72** | .16 |
| CSS-30 | .11 | .16 | .10 | **.65** | .11 | .25 | .15 | .16 | **.55** | .12 |
| CSS-31 | .17 | .06 | -.01 | **.44** | .24 | .10 | -.11 | .00 | **.49** | .18 |
| CSS-32 | -.28 | .17 | .07 | **.46** | .19 | .01 | .09 | -.01 | .39 | .37 |
| CSS-33 | .20 | -.01 | .07 | **.54** | .15 | .23 | .10 | -.02 | **.41** | .13 |
| CSS-34 | .21 | .00 | .09 | **.42** | .07 | .11 | .08 | .00 | **.50** | .21 |
| CSS-35 | .09 | .06 | .16 | **.48** | .11 | .04 | .38 | .09 | .32 | **.41** |
| CSS-36 | .10 | .04 | .20 | **.47** | .26 | .01 | .15 | .11 | .39 | **.46** |
| CSS = COVID Stress Scale Item; F1…F5 = Factors 1 through 5. Models fit using weighted least squares with mean and variance adjustment (WLSMV) estimation and targeted oblique rotation. The rotation targets for items not associated with a factor were set to 0. Factor loadings greater than λ = ±.40 presented in **bold.** Factor correlations ranged from *r* = .05 to .55 (average *r* = .28) in the Polish sample, and from *r* = .04 to .49 (average *r* = .31) in the Dutch sample. | | | | | | | | | | |
