# Supplementary material for: The Polish COVID Stress Scales: Considerations of psychometric functioning, measurement invariance, and validity
Source: PLoS One. 2021 Dec 1;16(12):e0260459. doi: 10.1371/journal.pone.0260459 (PMC8635383; doi:10.1371/journal.pone.0260459)
Supplement: S7 Table — CSS = COVID Stress Scale Item; F1…F6 = Factors 1 through 6. Models fit using weighted least squares with mean and variance adjustment (WLSMV) estimation and targeted oblique rotation. The rotation targets for items not associated with a factor were set to 0. Factor loadings greater than λ = ±.40 presented in bold. Factor correlations ranged from r = .05 to .55 (average r = .28) in the Polish sample, and from r = .04 to .49 (average r = .31) in the Dutch sample. (DOCX) [file pone.0260459.s009.docx]

| **S7 Table** | | | | | | | | | | | | |
| --- | --- | --- | --- | --- | --- | --- | --- | --- | --- | --- | --- | --- |
| *Factor Loadings From 6 Factor Exploratory Structural Equation Model* | | | | | | | | | | | | |
|  | Polish sample | | | | | | Dutch sample | | | | | |
|  | F1 | F2 | F3 | F4 | F5 | F6 | F1 | F2 | F3 | F4 | F5 | F6 |
| CSS-1 | .35 | -.13 | .14 | .36 | .25 | .03 | **.44** | -.01 | .08 | .25 | .21 | .00 |
| CSS-2 | **.48** | -.06 | .10 | .34 | .05 | .09 | **.48** | .13 | .08 | .19 | .24 | -.09 |
| CSS-3 | **.98** | .13 | .01 | -.14 | -.03 | .00 | **.65** | .12 | .06 | .10 | .02 | .07 |
| CSS-4 | **.59** | -.05 | .02 | .23 | .10 | .09 | **.79** | .05 | -.04 | .04 | .01 | .17 |
| CSS-5 | **.99** | .14 | -.09 | -.18 | -.09 | .07 | **.89** | .13 | .00 | -.01 | -.12 | .23 |
| CSS-6 | **.42** | .04 | .11 | .31 | .06 | .06 | **.46** | .21 | .03 | .24 | .13 | -.10 |
| CSS-7 | -.01 | **.90** | -.06 | .06 | .01 | .04 | .03 | **.82** | .01 | .00 | -.01 | .09 |
| CSS-8 | .03 | **.79** | .14 | -.07 | -.07 | .13 | .15 | **.92** | .01 | -.09 | .03 | -.08 |
| CSS-9 | .18 | **.76** | .05 | -.06 | -.03 | .13 | .18 | **.90** | -.06 | -.05 | .04 | -.13 |
| CSS-10 | -.08 | **.89** | .01 | .07 | .17 | -.09 | .07 | **.79** | .05 | .03 | .04 | .17 |
| CSS-11 | .15 | **.77** | .03 | .18 | .05 | -.09 | .10 | **.68** | .02 | .09 | .06 | -.02 |
| CSS-12 | -.01 | **.82** | .03 | .01 | .10 | -.09 | -.17 | **.66** | .16 | .08 | .05 | .09 |
| CSS-13 | .03 | -.02 | **.96** | -.13 | -.08 | .10 | .09 | .01 | **.82** | -.13 | .12 | -.10 |
| CSS-14 | .11 | -.02 | **.97** | -.05 | -.05 | .03 | .10 | -.03 | **.93** | .02 | .03 | -.12 |
| CSS-15 | .04 | -.01 | **.97** | -.02 | .00 | .01 | .04 | -.11 | **.99** | -.01 | .01 | -.04 |
| CSS-16 | -.17 | .18 | **.80** | -.01 | -.04 | .07 | -.22 | .09 | **.77** | .06 | -.15 | .22 |
| CSS-17 | -.11 | .12 | **.76** | .11 | .10 | -.14 | -.18 | .22 | **.51** | .22 | .01 | .19 |
| CSS-18 | .04 | -.10 | **.81** | .12 | .09 | -.06 | .02 | .03 | **.79** | .06 | .05 | -.03 |
| CSS-19 | .30 | -.09 | .20 | **.49** | .23 | -.06 | **.42** | -.07 | .27 | .38 | .05 | -.02 |
| CSS-20 | .15 | -.05 | .07 | **.73** | .08 | .02 | .20 | -.11 | .09 | **.66** | .05 | .06 |
| CSS-21 | .30 | -.11 | .05 | **.63** | .12 | -.01 | .38 | -.15 | .17 | **.53** | -.05 | .01 |
| CSS-22 | -.03 | .10 | -.03 | **.96** | -.07 | .13 | -.12 | .05 | -.07 | **.99** | .03 | -.05 |
| CSS-23 | -.10 | .11 | .03 | **.95** | -.11 | .13 | -.11 | .00 | -.08 | **.99** | .01 | .01 |
| CSS-24 | -.01 | .23 | .07 | **.76** | -.01 | .01 | -.06 | .15 | .04 | **.70** | -.04 | .13 |
| CSS-25 | .07 | .05 | -.02 | .04 | **.69** | .16 | -.10 | .00 | .11 | -.07 | **.85** | .13 |
| CSS-26 | -.18 | .04 | .14 | .00 | **.69** | .20 | -.18 | .16 | .16 | -.08 | **.73** | .18 |
| CSS-27 | .12 | .03 | -.03 | -.08 | **.90** | -.01 | .06 | -.10 | -.24 | .12 | **.85** | -.04 |
| CSS-28 | .06 | -.03 | .02 | -.02 | **.89** | .03 | .10 | .02 | .02 | .01 | **.78** | .04 |
| CSS-29 | -.05 | .07 | -.04 | .02 | **.88** | .07 | .03 | -.01 | -.01 | .00 | **.86** | .05 |
| CSS-30 | .00 | .13 | .07 | -.04 | **.79** | .01 | .07 | .14 | .12 | .08 | **.68** | .01 |
| CSS-31 | .18 | .00 | -.08 | .01 | .06 | **.61** | .18 | -.22 | .00 | -.05 | .17 | **.54** |
| CSS-32 | -.13 | .11 | -.03 | -.16 | -.05 | **.70** | .05 | -.03 | -.03 | .02 | -.10 | **.80** |
| CSS-33 | .06 | -.06 | -.01 | .08 | .36 | **.41** | .21 | .04 | -.03 | .04 | .24 | .36 |
| CSS-34 | .02 | -.05 | .01 | .20 | .10 | **.49** | .17 | -.02 | -.02 | -.04 | .24 | **.49** |
| CSS-35 | -.03 | .00 | .07 | .13 | .04 | **.64** | -.05 | .29 | .04 | .10 | .11 | **.55** |
| CSS-36 | .12 | -.03 | .12 | -.03 | .04 | **.68** | -.05 | .02 | .08 | .08 | .04 | **.73** |
| CSS = COVID Stress Scale Item; F1…F6 = Factors 1 through 6. Models fit using weighted least squares with mean and variance adjustment (WLSMV) estimation and targeted oblique rotation. The rotation targets for items not associated with a factor were set to 0. Factor loadings greater than λ = ±.40 presented in **bold.** Factor correlations ranged from *r* = .05 to .55 (average *r* = .28) in the Polish sample, and from *r* = .04 to .49 (average *r* = .31) in the Dutch sample. | | | | | | | | | | | | |
