# Supplementary material for: The Polish COVID Stress Scales: Considerations of psychometric functioning, measurement invariance, and validity
Source: PLoS One. 2021 Dec 1;16(12):e0260459. doi: 10.1371/journal.pone.0260459 (PMC8635383; doi:10.1371/journal.pone.0260459)
Supplement: S8 Table — CSS = COVID Stress Scale Item; F1…F5 = Factors 1 through 5. Models fit using weighted least squares with mean and variance adjustment (WLSMV) estimation. The rotation targets for items not associated with a factor were set to 0. Factor correlations ranged from r = .31 to .82 (average r = .54) in the Polish sample, and from r = .43 to .75 (average r = .57) in the Dutch sample. Model fit for the Polish sample was: χ2 = 3447.67, df = 584, p < .01; RMSEA = .094; SRMR = .08; CFI = .919; TLI = .912. Model fit for the Dutch sample was: χ2 = 1720.01, df = 585, p < .01; RMSEA = .071; SRMR = .076; CFI = .937; TLI = .933. Dutch sample has one extra degree of freedom because the factor loading for CSS-10 was fixed to .98 to avoid convergence with negative residual variances. (DOCX) [file pone.0260459.s010.docx]

| **S8 Table** | | | | | | | | | | |
| --- | --- | --- | --- | --- | --- | --- | --- | --- | --- | --- |
| *Factor Loadings From 5 Factor Correlated Factors Model* | | | | | | | | | | |
|  | Polish sample | | | | | Dutch sample | | | | |
|  | F1 | F2 | F3 | F4 | F5 | F1 | F2 | F3 | F4 | F5 |
| CSS-1 | .87 | -- | -- | -- | -- | .75 | -- | -- | -- | -- |
| CSS-2 | .80 | -- | -- | -- | -- | .79 | -- | -- | -- | -- |
| CSS-3 | .83 | -- | -- | -- | -- | .75 | -- | -- | -- | -- |
| CSS-4 | .79 | -- | -- | -- | -- | .79 | -- | -- | -- | -- |
| CSS-5 | .81 | -- | -- | -- | -- | .82 | -- | -- | -- | -- |
| CSS-6 | .75 | -- | -- | -- | -- | .73 | -- | -- | -- | -- |
| CSS-7 | -- | .85 | -- | -- | -- | -- | .85 | -- | -- | -- |
| CSS-8 | -- | .85 | -- | -- | -- | -- | .86 | -- | -- | -- |
| CSS-9 | -- | .86 | -- | -- | -- | -- | .81 | -- | -- | -- |
| CSS-10 | -- | .90 | -- | -- | -- | -- | .98 | -- | -- | -- |
| CSS-11 | -- | .91 | -- | -- | -- | -- | .82 | -- | -- | -- |
| CSS-12 | -- | .79 | -- | -- | -- | -- | .81 | -- | -- | -- |
| CSS-13 | -- | -- | .86 | -- | -- | -- | -- | .80 | -- | -- |
| CSS-14 | -- | -- | .96 | -- | -- | -- | -- | .94 | -- | -- |
| CSS-15 | -- | -- | .97 | -- | -- | -- | -- | .93 | -- | -- |
| CSS-16 | -- | -- | .79 | -- | -- | -- | -- | .74 | -- | -- |
| CSS-17 | -- | -- | .82 | -- | -- | -- | -- | .87 | -- | -- |
| CSS-18 | -- | -- | .92 | -- | -- | -- | -- | .86 | -- | -- |
| CSS-19 | .92 | -- | -- | -- | -- | .80 | -- | -- | -- | -- |
| CSS-20 | .88 | -- | -- | -- | -- | .78 | -- | -- | -- | -- |
| CSS-21 | .88 | -- | -- | -- | -- | .72 | -- | -- | -- | -- |
| CSS-22 | .91 | -- | -- | -- | -- | .89 | -- | -- | -- | -- |
| CSS-23 | .89 | -- | -- | -- | -- | .87 | -- | -- | -- | -- |
| CSS-24 | .82 | -- | -- | -- | -- | .75 | -- | -- | -- | -- |
| CSS-25 | -- | -- | -- | .89 | -- | -- | -- | -- | .85 | -- |
| CSS-26 | -- | -- | -- | .84 | -- | -- | -- | -- | .88 | -- |
| CSS-27 | -- | -- | -- | .89 | -- | -- | -- | -- | .71 | -- |
| CSS-28 | -- | -- | -- | .93 | -- | -- | -- | -- | .88 | -- |
| CSS-29 | -- | -- | -- | .91 | -- | -- | -- | -- | .87 | -- |
| CSS-30 | -- | -- | -- | .85 | -- | -- | -- | -- | .95 | -- |
| CSS-31 | -- | -- | -- | -- | .65 | -- | -- | -- | -- | .82 |
| CSS-32 | -- | -- | -- | -- | .33 | -- | -- | -- | -- | .55 |
| CSS-33 | -- | -- | -- | -- | .80 | -- | -- | -- | -- | .73 |
| CSS-34 | -- | -- | -- | -- | .71 | -- | -- | -- | -- | .69 |
| CSS-35 | -- | -- | -- | -- | .73 | -- | -- | -- | -- | .90 |
| CSS-36 | -- | -- | -- | -- | .78 | -- | -- | -- | -- | .75 |
| CSS = COVID Stress Scale Item; F1…F5 = Factors 1 through 5. Models fit using weighted least squares with mean and variance adjustment (WLSMV) estimation. The rotation targets for items not associated with a factor were set to 0. Factor correlations ranged from *r* = .31 to .82 (average *r* = .54) in the Polish sample, and from *r* = .43 to .75 (average *r* = .57) in the Dutch sample. Model fit for the Polish sample was: χ^2^ = 3447.67, df = 584, *p* < .01; RMSEA = .094; SRMR = .08; CFI = .919; TLI = .912. Model fit for the Dutch sample was: χ^2^ = 1720.01, df = 585, *p* < .01; RMSEA = .071; SRMR = .076; CFI = .937; TLI = .933. Dutch sample has one extra degree of freedom because the factor loading for CSS-10 was fixed to .98 to avoid convergence with negative residual variances. | | | | | | | | | | |
