# Supplementary material for: The Polish COVID Stress Scales: Considerations of psychometric functioning, measurement invariance, and validity
Source: PLoS One. 2021 Dec 1;16(12):e0260459. doi: 10.1371/journal.pone.0260459 (PMC8635383; doi:10.1371/journal.pone.0260459)
Supplement: S9 Table — *** p < .001. (DOCX) [file pone.0260459.s011.docx]

| **S9 Table**  *Reliability of the Polish COVID-Stress Scales (CSS-PL) and Correlations among the CSS-PL Scales* | | | | | |
| --- | --- | --- | --- | --- | --- |
| COVID-Stress Scales | McDonald’s *ω* | 1 | 2 | 3 | 4 |
| 1. COVID danger and contamination | .94 | - |  |  |  |
| 2. COVID socioeconomic consequences | .91 | .26*** | - |  |  |
| 3. COVID xenophobia | .93 | .52*** | .42*** | - |  |
| 4. COVID traumatic stress symptoms | .93 | .63*** | .34*** | .48*** | - |
| 5. COVID compulsive checking | .80 | .56*** | .30*** | .41*** | .69*** |
| *Note.* *** *p* < .001. | | | | | |
