# Supplementary material for: The Polish COVID Stress Scales: Considerations of psychometric functioning, measurement invariance, and validity
Source: PLoS One. 2021 Dec 1;16(12):e0260459. doi: 10.1371/journal.pone.0260459 (PMC8635383; doi:10.1371/journal.pone.0260459)
Supplement: S10 Table — DC = Danger-Contamination Scale; SES = Socioeconomic Consequences Scale; XN = Xenophobia Scale; TR = Traumatic Stress Scale; CK = Checking Scale; I χ2 = overall test of measurement invariance across items; a χ2 = test of measurement invariance in item discrimination values; b χ2 = test of measurement invariance in item difficulty parameters. Chi square values from specific measurement invariance tests presented in cells; bold denotes a statistically significant chi square value at p < .05. Significant values here suggest that an item or item parameter may be non-invariant across time (i.e., significant differences across time).The initial “All Items” sweep was conducted to identify anchor items and items that may demonstrate non-invariance. This process may over-identify non-invariance however, and so more targeted follow-up tests were conducted using the items and parameters that demonstrated invariance at a previous stage as anchors. The exception was that the presence of non-invariance in the discrimination value suggests that the whole item should be treated as functioning differently across time, even if there is no evidence for non-invariance in the difficulty parameters (i.e., equal difficulty in the absence of equal discrimination values is not particularly meaningful). In all models Time 1 was treated as the reference group (factor mean and variance fixed to 0 and 1, respectively) and Time 2 was treated as the focal group (factor and variance freely estimated). (DOCX) [file pone.0260459.s012.docx]

| **S10 Table** | | | | | | | | | | | | |
| --- | --- | --- | --- | --- | --- | --- | --- | --- | --- | --- | --- | --- |
| *Results From COVID Stress Scale Measurement Invariance Tests Across Times 1 and 2* | | | | | | | | | | | | |
|  | All Items | | | Candidate Items 1 | | | Candidate Items 2 | | | Candidate Items 3 | | |
|  | I χ^2^ | *a* χ^2^ | *b* χ^2^ | I χ^2^ | *a* χ^2^ | *b* χ^2^ | I χ^2^ | *a* χ^2^ | *b* χ^2^ | I χ^2^ | *a* χ^2^ | *b* χ^2^ |
| DC-1 | 5.60 | .70 | 4.90 | -- | -- | -- | -- | -- | -- | -- | -- | -- |
| DC-2 | 8.30 | **4.00** | 4.30 | 8.40 | **5.10** | 3.30 | 7.40 | 2.30 | 5.00 | -- | -- | -- |
| DC-3 | **12.10** | 1.70 | **10.40** | **17.40** | 2.60 | **14.80** | **14.30** | 0.00 | **14.30** | **14.40** | .00 | **14.40** |
| DC-4 | 10.60 | .70 | **10.00** | **13.90** | 1.30 | **12.70** | **12.90** | 0.00 | **12.90** | **13.10** | .00 | **13.10** |
| DC-5 | **14.30** | 2.10 | **12.20** | **21.30** | 3.10 | **18.20** | **17.10** | 0.00 | **17.10** | **17.40** | .00 | **17.40** |
| DC-6 | 9.70 | 1.50 | 8.20 | -- | -- | -- | -- | -- | -- | -- | -- | -- |
| DC-7 | 2.40 | .20 | 2.10 | -- | -- | -- | -- | -- | -- | -- | -- | -- |
| DC-8 | 1.80 | .30 | 1.60 | -- | -- | -- | -- | -- | -- | -- | -- | -- |
| DC-9 | 4.50 | .60 | 3.90 | -- | -- | -- | -- | -- | -- | -- | -- | -- |
| DC-10 | 2.00 | .00 | 2.00 | -- | -- | -- | -- | -- | -- | -- | -- | -- |
| DC-11 | 5.00 | .10 | 4.90 | -- | -- | -- | -- | -- | -- | -- | -- | -- |
| DC-12 | 1.80 | .20 | 1.60 | -- | -- | -- | -- | -- | -- | -- | -- | -- |
| SES-1 | 5.20 | 2.70 | 2.50 | -- | -- | -- | -- | -- | -- | -- | -- | -- |
| SES-2 | 5.00 | 1.70 | 3.30 | -- | -- | -- | -- | -- | -- | -- | -- | -- |
| SES-3 | 1.00 | .00 | 1.00 | -- | -- | -- | -- | -- | -- | -- | -- | -- |
| SES-4 | 5.20 | .10 | 5.10 | -- | -- | -- | -- | -- | -- | -- | -- | -- |
| SES-5 | 4.60 | .10 | 4.50 | -- | -- | -- | -- | -- | -- | -- | -- | -- |
| SES-6 | 3.70 | .00 | 3.60 | -- | -- | -- | -- | -- | -- | -- | -- | -- |
| XN-1 | **11.50** | 3.40 | 8.10 | **24.60** | **12.60** | **12.10** | -- | -- | -- | -- | -- | -- |
| XN-2 | 4.60 | .00 | 4.60 | -- | -- | -- | -- | -- | -- | -- | -- | -- |
| XN-3 | 4.90 | .90 | 4.10 | -- | -- | -- | -- | -- | -- | -- | -- | -- |
| XN-4 | 3.00 | .40 | 2.60 | -- | -- | -- | -- | -- | -- | -- | -- | -- |
| XN-5 | 7.80 | .30 | 7.50 | -- | -- | -- | -- | -- | -- | -- | -- | -- |
| XN-6 | 6.30 | 2.50 | 3.90 | -- | -- | -- | -- | -- | -- | -- | -- | -- |
| TR-1 | 10.50 | 1.10 | 9.40 | -- | -- | -- | -- | -- | -- | -- | -- | -- |
| TR-2 | 6.20 | 1.50 | 4.70 | -- | -- | -- | -- | -- | -- | -- | -- | -- |
| TR-3 | 1.30 | .00 | 1.30 | -- | -- | -- | -- | -- | -- | -- | -- | -- |
| TR-4 | 6.90 | .70 | 6.20 | -- | -- | -- | -- | -- | -- | -- | -- | -- |
| TR-5 | 4.40 | 1.20 | 3.30 | -- | -- | -- | -- | -- | -- | -- | -- | -- |
| TR-6 | 2.60 | 1.50 | 1.00 | -- | -- | -- | -- | -- | -- | -- | -- | -- |
| CK-1 | 5.20 | 2.40 | 2.80 | -- | -- | -- | -- | -- | -- | -- | -- | -- |
| CK-2 | 7.40 | **5.10** | 2.30 | 7.10 | **4.30** | 2.70 | **7.10** | **4.30** | 2.80 | -- | -- | -- |
| CK-3 | 3.10 | .60 | 2.40 | -- | -- | -- | -- | -- | -- | -- | -- | -- |
| CK-4 | **12.90** | .20 | 12.70 | **12.90** | .50 | **12.40** | **12.10** | .00 | **12.10** | -- | -- | -- |
| CK-5 | 2.30 | 1.10 | 1.20 | -- | -- | -- | -- | -- | -- | -- | -- | -- |
| CK-6 | 2.90 | .00 | 2.80 | -- | -- | -- | -- | -- | -- | -- | -- | -- |
| DC = Danger-Contamination Scale; SES = Socioeconomic Consequences Scale; XN = Xenophobia Scale; TR = Traumatic Stress Scale; CK = Checking Scale; I χ^2^ = overall test of measurement invariance across items; *a* χ^2^ = test of measurement invariance in item discrimination values; *b* χ^2^ = test of measurement invariance in item difficulty parameters. Chi square values from specific measurement invariance tests presented in cells; bold denotes a statistically significant chi square value at p < .05. Significant values here suggest that an item or item parameter may be non-invariant across time (i.e., significant differences across time).The initial “All Items” sweep was conducted to identify anchor items and items that may demonstrate non-invariance. This process may over-identify non-invariance however, and so more targeted follow-up tests were conducted using the items and parameters that demonstrated invariance at a previous stage as anchors. The exception was that the presence of non-invariance in the discrimination value suggests that the whole item should be treated as functioning differently across time, even if there is no evidence for non-invariance in the difficulty parameters (i.e., equal difficulty in the absence of equal discrimination values is not particularly meaningful). In all models Time 1 was treated as the reference group (factor mean and variance fixed to 0 and 1, respectively) and Time 2 was treated as the focal group (factor and variance freely estimated). | | | | | | | | | | | | |
