# Supplementary material for: The Polish COVID Stress Scales: Considerations of psychometric functioning, measurement invariance, and validity
Source: PLoS One. 2021 Dec 1;16(12):e0260459. doi: 10.1371/journal.pone.0260459 (PMC8635383; doi:10.1371/journal.pone.0260459)
Supplement: S11 Table — DC = Danger-Contamination Scale; SES = Socioeconomic Consequences Scale; XN = Xenophobia Scale; TR = Traumatic Stress Scale; CK = Checking Scale; a = item discrimination; b1…b4 = item difficulty parameters; dMiAssumed = Cohen’s d for the scale mean difference across time with measurement invariance assumed (i.e., all item parameters constrained to equality); dMiModeled = Cohen’s d for the scale mean difference across time with measurement invariance modeled (i.e., only invariant item parameters constrained across time). Items that demonstrated non-invariance are presented in bold. Cohen’s ds were computed with Time 1 as the reference group (i.e., positive values denote that scores were higher at Time 2). (DOCX) [file pone.0260459.s013.docx]

| **S11 Table** | | | | | | | | | | | | |
| --- | --- | --- | --- | --- | --- | --- | --- | --- | --- | --- | --- | --- |
| *Results From COVID Stress Scale Item Response Models With Parameter Constraints Across Times 1 and 2 Supported by DIF Tests* | | | | | | | | | | | | |
|  | Time 1 | | | | | Time 2 | | | | |  | |
|  | *a* | *b*_1_ | *b*_2_ | *b*_3_ | *b*_4_ | *a* | *b*_1_ | *b*_2_ | *b*_3_ | *b*_4_ | *d*_MiAssumed_ | *d*_MiModeled_ |
| DC-1 | 3.18 | -1.03 | -.14 | .68 | 1.67 | 3.18 | -1.03 | -.14 | .68 | 1.67 | -- | -- |
| DC-2 | 2.79 | -.88 | -.08 | .63 | 1.79 | 2.79 | -.98 | -.02 | .71 | 2.06 | -- | -- |
| **DC-3** | **1.46** | **-1.87** | **-1.15** | **-.39** | **1.07** | **1.46** | **-1.86** | **-.92** | **.07** | **1.41** | -- | -- |
| **DC-4** | **2.38** | **-1.21** | **-.50** | **.34** | **1.47** | **2.38** | **-1.34** | **-.28** | **.53** | **1.76** | -- | -- |
| **DC-5** | **1.34** | **-1.98** | **-1.31** | **-.54** | **1.02** | **1.34** | **-1.95** | **-.98** | **-.05** | **1.54** | -- | -- |
| DC-6 | 2.31 | -.92 | -.07 | .82 | 1.95 | 2.31 | -.92 | -.07 | .82 | 1.95 | -- | -- |
| DC-7 | 4.56 | -.84 | .16 | .76 | 1.50 | 4.56 | -.84 | .16 | .76 | 1.50 | -- | -- |
| DC-8 | 3.97 | -.67 | .14 | .78 | 1.63 | 3.97 | -.67 | .14 | .78 | 1.63 | -- | -- |
| DC-9 | 3.89 | -1.05 | -.18 | .37 | 1.22 | 3.89 | -1.05 | -.18 | .37 | 1.22 | -- | -- |
| DC-10 | 3.51 | -.31 | .44 | 1.22 | 1.95 | 3.51 | -.31 | .44 | 1.22 | 1.95 | -- | -- |
| DC-11 | 2.87 | -.09 | .77 | 1.51 | 2.21 | 2.87 | -.09 | .77 | 1.51 | 2.21 | -- | -- |
| DC-12 | 2.53 | .24 | 1.1 | 1.76 | -- | 2.53 | .24 | 1.1 | 1.76 | -- | -- | -- |
| Scale *d* | -- | -- | -- | -- | -- | -- | -- | -- | -- | -- | -.33 | -.29 |
| SES-1 | 3.97 | .34 | .98 | 1.78 | -- | 3.97 | .34 | .98 | 1.78 | -- | -- | -- |
| SES-2 | 3.29 | .42 | 1.15 | 2.03 | -- | 3.29 | .42 | 1.15 | 2.03 | -- | -- | -- |
| SES-3 | 2.45 | -.10 | .84 | 1.70 | 2.51 | 2.45 | -.10 | .84 | 1.70 | 2.51 | -- | -- |
| SES-4 | 3.55 | .68 | 1.33 | 2.07 | -- | 3.55 | .68 | 1.33 | 2.07 | -- | -- | -- |
| SES-5 | 2.60 | .18 | 1.11 | 2.04 | -- | 2.60 | .18 | 1.11 | 2.04 | -- | -- | -- |
| SES-6 | 2.61 | .49 | 1.29 | 1.86 | -- | 2.61 | .49 | 1.29 | 1.86 | -- | -- | -- |
| Scale *d* | -- | -- | -- | -- | -- | -- | -- | -- | -- | -- | -.39 | -.39 |
| **XN-1** | **3.55** | **.35** | **.98** | **1.55** | **2.25** | **5.34** | **.22** | **.92** | **1.58** | **2.17** | -- | -- |
| XN-2 | 5.90 | .04 | .85 | 1.47 | 2.19 | 5.90 | .04 | .85 | 1.47 | 2.19 | -- | -- |
| XN-3 | 9.91 | .28 | .95 | 1.48 | 2.12 | 9.91 | .28 | .95 | 1.48 | 2.12 | -- | -- |
| XN-4 | 2.41 | .78 | 1.55 | 2.27 | 2.88 | 2.41 | .78 | 1.55 | 2.27 | 2.88 | -- | -- |
| XN-5 | 2.50 | .71 | 1.40 | 2.01 | -- | 2.50 | .71 | 1.40 | 2.01 | -- | -- | -- |
| XN-6 | 3.39 | -.02 | .85 | 1.46 | 2.10 | 3.39 | -.02 | .85 | 1.46 | 2.10 | -- | -- |
| Scale *d* | -- | -- | -- | -- | -- | -- | -- | -- | -- | -- | -.03 | -.03 |
| TR-1 | 3.09 | .13 | .69 | 1.40 | 2.43 | 3.09 | .13 | .69 | 1.40 | 2.43 | -- | -- |
| TR-2 | 2.31 | .70 | 1.37 | 2.04 | -- | 2.31 | .70 | 1.37 | 2.04 | -- | -- | -- |
| TR-3 | 3.60 | -.61 | .26 | .92 | 1.93 | 3.60 | -.61 | .26 | .92 | 1.93 | -- | -- |
| TR-4 | 4.71 | -.19 | .46 | 1.11 | 1.99 | 4.71 | -.19 | .46 | 1.11 | 1.99 | -- | -- |
| TR-5 | 4.51 | .09 | .76 | 1.41 | 2.16 | 4.51 | .09 | .76 | 1.41 | 2.16 | -- | -- |
| TR-6 | 2.66 | .20 | .83 | 1.41 | 2.3 | 2.66 | .20 | .83 | 1.41 | 2.30 | -- | -- |
| Scale *d* | -- | -- | -- | -- | -- | -- | -- | -- | -- | -- | -.31 | -.31 |
| CK-1 | 1.64 | -1.79 | -.72 | .16 | 1.37 | 1.64 | -1.79 | -.72 | .16 | 1.37 | -- | -- |
| **CK-2** | **.94** | **-.57** | **.97** | **2.15** | **3.87** | **1.44** | **-.33** | **.83** | **1.75** | **3.28** | -- | -- |
| CK-3 | 1.70 | -.37 | .60 | 1.42 | 2.66 | 1.70 | -.37 | .60 | 1.42 | 2.66 | -- | -- |
| **CK-4** | **1.55** | **-.67** | **.14** | **1.16** | **2.38** | **1.55** | **-1.02** | **.20** | **1.26** | **3.00** | -- | -- |
| CK-5 | 1.73 | .39 | 1.38 | 2.26 | -- | 1.73 | .39 | 1.38 | 2.26 | -- | -- | -- |
| CK-6 | 2.59 | -.87 | -.12 | .78 | 1.84 | 2.59 | -.87 | -.12 | .78 | 1.84 | -- | -- |
| Scale *d* | -- | -- | -- | -- | -- | -- | -- | -- | -- | -- | -.49 | -.49 |
| DC = Danger-Contamination Scale; SES = Socioeconomic Consequences Scale; XN = Xenophobia Scale; TR = Traumatic Stress Scale; CK = Checking Scale; *a* = item discrimination; *b*_1_…*b*_4_ = item difficulty parameters; *d*_MiAssumed_ = Cohen’s *d* for the scale mean difference across time with measurement invariance assumed (i.e., all item parameters constrained to equality); *d*_MiModeled_ = Cohen’s *d* for the scale mean difference across time with measurement invariance modeled (i.e., only invariant item parameters constrained across time). Items that demonstrated non-invariance are presented in **bold**. Cohen’s *d*s were computed with Time 1 as the reference group (i.e., positive values denote that scores were higher at Time 2). | | | | | | | | | | | | |
