# Supplementary material for: The Polish COVID Stress Scales: Considerations of psychometric functioning, measurement invariance, and validity
Source: PLoS One. 2021 Dec 1;16(12):e0260459. doi: 10.1371/journal.pone.0260459 (PMC8635383; doi:10.1371/journal.pone.0260459)
Supplement: S12 Table — Sample of 264 participants at Time 2. Correlations ≥ .30 in bold. (DOCX) [file pone.0260459.s014.docx]

| **S12 Table**  *Tests of Discriminant Validity: Comparison Of the CSS-PL Correlations with Current Anxiety and Depression* | | | | | |
| --- | --- | --- | --- | --- | --- |
|  | COVID-Stress Scales | | | | |
| Variables | COVID  danger and contamination | COVID socioeconomic consequences | COVID xenophobia | COVID traumatic stress symptoms | COVID compulsive checking |
| Current anxiety | .**44** | .17 | .27 | **.53** | .20 |
| Current depression | **.35** | .17 | .**34** | .**36** | .16 |
| Significance of difference  between rs: Z | 1.22  *p* = .222 | 0.00  *p* = 1.00 | -0.88  *p* = .378 | 2.44  *p* = .015 | 0.47  *p* = .637 |
| *Note.* Sample of 264 participants at Time 2. Correlations ≥ .30 in bold. | | | | | |
