# Supplementary material for: The Polish COVID Stress Scales: Considerations of psychometric functioning, measurement invariance, and validity
Source: PLoS One. 2021 Dec 1;16(12):e0260459. doi: 10.1371/journal.pone.0260459 (PMC8635383; doi:10.1371/journal.pone.0260459)
Supplement: S13 Table — General distress = correlations of the CSS-PL with the measures of general anxiety, depression, and trait measures of health anxiety and OC symptoms at Time 2. Sample of 264 participants at Time 2. Correlations ≥ .30 in bold. (DOCX) [file pone.0260459.s015.docx]

| **S13 Table**  *Tests of Discriminant Validity: Comparison of the Mean Correlations For Distress Measures Versus Correlation with General Xenophobia* | | | | | |
| --- | --- | --- | --- | --- | --- |
|  | COVID-Stress Scales | | | | |
| Variables | COVID  danger and contamination | COVID socioeconomic consequences | COVID xenophobia | COVID traumatic stress symptoms | COVID compulsive checking |
| Mean r: general distress | .**45** | .17 | **.32** | **.47** | .26 |
| r: xenophobia | -.05 | .10 | .25 | .00 | .04 |
| Significance of difference  between rs: Z | 4.97  *p* ≤ .001 | 0.82  *p* = .415 | 0.87  *p* = .384 | 5.83  *p* ≤ .001 | 2.58  *p* ≤ .001 |
| *Note.* General distress = correlations of the CSS-PL with the measures of general anxiety, depression, and trait measures of health anxiety and OC symptoms at Time 2. Sample of 264 participants at Time 2. Correlations ≥ .30 in bold. | | | | | |
